# Supplementary material for: Reconstitution of monoterpene indole alkaloid biosynthesis in genome engineered Nicotiana benthamiana
Source: Commun Biol. 2022 Sep 10;5:949. doi: 10.1038/s42003-022-03904-w (PMC9464250; doi:10.1038/s42003-022-03904-w)
Supplement: Supplementary file 1 — Supplementary Information [file 42003_2022_3904_MOESM1_ESM.pdf]

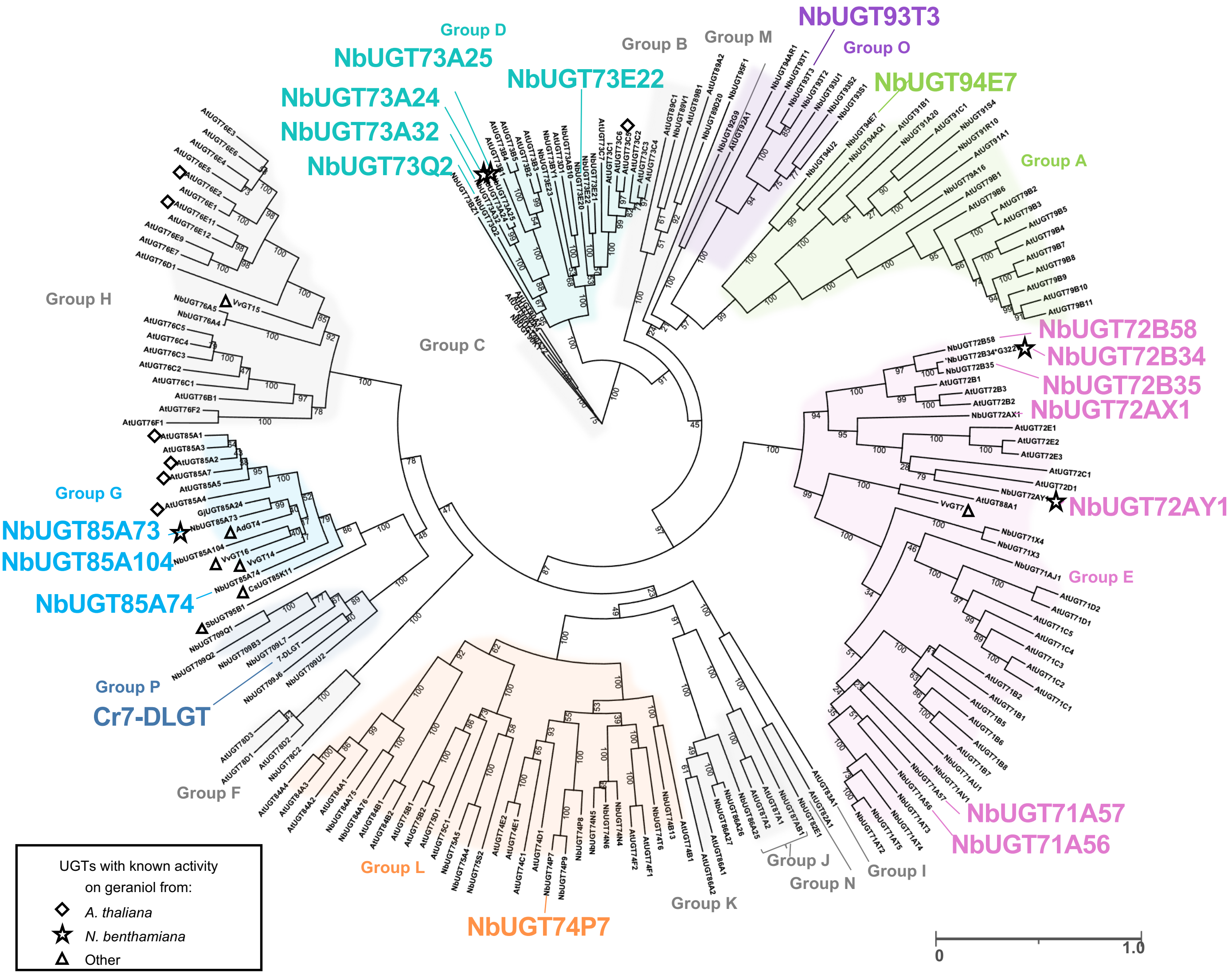

Supplementary Figure 1. Maximum likelihood (RAxML) phylogenetic comparison of 193 Family 1 UDP-glycosyltransferases (UGTs). Sequences are from *N. benthamiana*, *A. thaliana* plus nine UGTs previously shown to be active on geraniol or iridoid substrates. Groups A-P are annotated according to the nomenclature used by Caputi et al 2011. Labeled taxa indicate enzymes in which Cas9-mediated targeted mutations were subsequently introduced. Numbers at nodes are bootstrap support. Scale bar indicates the number of substitutions per site.

a

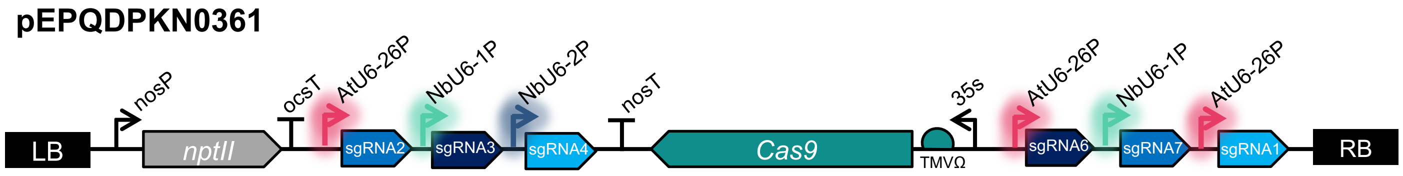

b

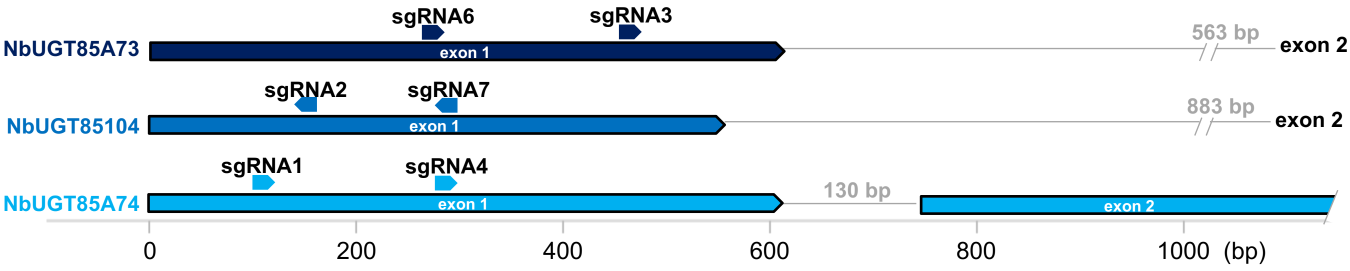

c

NbUGT85A73			
Plant line	sgRNA6<br>GAGAAATGGGTTCTGT-TGAAGGG	sgRNA3<br>ACCCGTCTCGTGCATCG-TGTCGG	T-DNA
0361-34-05	**bi-allelic**<br>CGTGGCCCTGATTCTCTTCAAGGG<br>CGTGGCCCTGAT-----TCAAGGG<br>frameshift at aa63 and stop codon at aa71	**homozygous**<br>ACCCGTCTCGTGCATCGTTGTCGG<br>sequence remains out of frame	-
0361-34-08	**homozygous**<br>CGTGGCCCTGAT-----TCAAGGG<br>frameshift resulting in incorrect sequence from aa62 -125	**homozygous**<br>ACCCGTCTCGTGCATCGTTGTCGG<br>seqeunce back in frame from S126	+

NbUGT85A104			
Plant line	sgRNA2<br>CCTCCATAT-ACGAGGCTTTCATA	sgRNA7<br>CCCTC-TAACGATGACGCGACACA	T-DNA
0361-34-05	**homozygous**<br>CCTCCATATAACGAGGCTTTCATA<br>frameshift at aa40 and stop codon at aa50	**homozygous**<br>CCCTCTTAACGATGACGCGACACA<br>sequence remains out of frame	-
0361-34-08	**homozygous**<br>CCTCCATATAACGAGGCTTTCATA<br>frameshift at aa40 and stop codon at aa50	**homozygous**<br>CCCTCTTAACGATGACGCGACACA<br>sequence remains out of frame	+

NbUGT85A74			
Plant line	sgRNA1<br>GAGAAATGGGTTCTGTT-GAAGGG	sgRNA4<br>AGAGGACCTTATTCTCTTAAAGG	T-DNA
0361-34-05	**bi-allelic**<br>GAGAAATGGGTTCTGTTCGAAGGG<br>GAGAAATGGGTTCTGTTAGAAGGG<br>frameshift at aa5 and stop codon at aa47	**wild type**<br>AGAGGACCTTATTCTCTTAAAGG<br>n/a	-
0361-34-08	**homozygous**<br>GAGAAATGGGTTCTGTTAGAAGGG<br>frameshift at aa5 and stop codon at aa47	**wild type**<br>AGAGGACCTTATTCTCTTAAAGG<br>n/a	+

Supplementary Figure 2. Cas9-mediated mutagenesis of three Group G UGTs (a) Schematic showing construct design (b) locations of sgRNAs (c) sequences of targets and genotypes of T1 plants

a

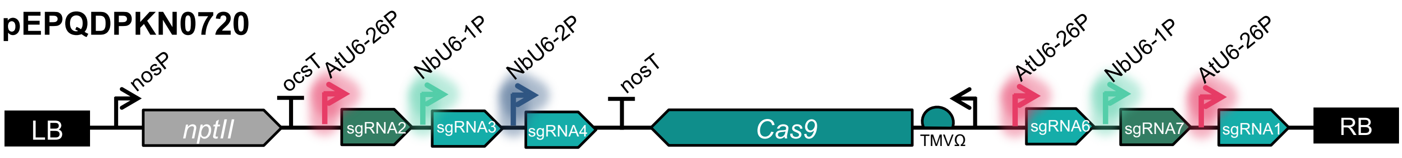

b

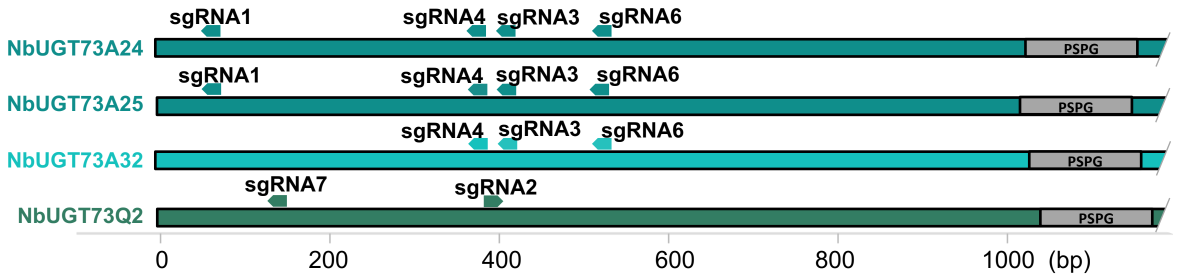

c

NbUGT73A24

Plant line	sgRNA1<br>CCTACACTAGACATGGCGAAGCT	sgRNA4<br>CCTTGGACTACTGATTCTGCAGC	sgRNA3<br>CCGAGAATAGTTTTCCATGGCAC	sgRNA6<br>CCGAA-TTTGCCTCACGAAATTAA	T-DNA
0720-06-01	**homozygous**<br>CCTACA-----------------<br>319 bp deletion from sgRNA1 to sgRNA4 deleting aa21-126 and introducing frameshift	**homozygous**<br>-------------ATTCTGCAGC	**homozygous**<br>CCGAG---AGTTTTCCATGGCAC<br>sequence remains out of frame	**homozygous**<br>CCGAATTTTGCCTCACGAAATTAA<br>sequence remains out of frame	-
0720-06-03	**homozygous**<br>CCTACA-----------------<br>319 bp deletion from sgRNA1 to sgRNA4 deleting aa21-126 and introducing frameshift	**homozygous**<br>-------------ATTCTGCAGC	**homozygous**<br>CCGAG---AGTTTTCCATGGCAC<br>sequence remains out of frame	**homozygous**<br>CCGAATTTTGCCTCACGAAATTAA<br>sequence remains out of frame	+

NbUGT73A25

Plant line	sgRNA1<br>CCTACA-CTAGACATGGCGAAGCT	sgRNA4<br>CCTTGGACTACTGATTCTGCAGC<br>Mismatch in sgRNA	sgRNA3<br>CCGAGAATAGTTTTCCATGGCACAAGCTTC	sgRNA6<br>CCGAA-TTTGCCTCACGAAATTAA	T-DNA
0720-06-01	**bi-allelic**<br>CCTACACCTAGACATGGCGAAGCT<br>CCTACA---AGACATGGCGAAGCT<br>frameshift from aa21 and stop codon at aa 32	**wild type**<br>CCTTGGACTACTGATAGTGCAGC<br>sequence remains out of frame	**bi-allelic**<br>CCGAGAA------TCCATGGCACAAGCTTC<br>CCGAGA---------------------TTC<br>sequence remains out of frame	**homozygous**<br>CCGAATTTTGCCTCACGAAATTAA<br>sequence remains out of frame	-
0720-06-03	**homozygous**<br>CCTACACCTAGACATGGCGAAGCT<br>frameshift from aa21 and stop codon at aa 33	**wild type**<br>CCTTGGACTACTGATAGTGCAGC<br>sequence remains out of frame	**homozygous**<br>CCGAGAA------TCCATGGCACAAGCTTC<br>sequence remains out of frame	**homozygous**<br>CCGAATTTTGCCTCACGAAATTAA<br>sequence remains out of frame	+

NbUGT73A32

Plant line	sgRNA4<br>CCTTGGACTACTGATTCTGCAGC	sgRNA3<br>CCGAGAATAGTTTTTCATGGTAC<br>Mismatch in sgRNA	sgRNA6<br>CCGAATTTGCCTCACGAAATTAA<br>Mismatch in sgRNA	T-DNA
0720-06-01	**homozygous**<br>CCTTG-------GATTCTGCAGC<br>frameshift from aa125 and stop codon at aa135	**wild type**<br>CCAAGAATAGTTTTTCATGGTAC<br>sequence remains out of frame	**wild type**<br>CCTAATTTGCCTCACGAAATCAA<br>sequence remains out of frame	-
0720-06-03	**homozygous**<br>CCTTG-------GATTCTGCAGC<br>frameshift from aa125 and stop codon at aa135	**wild type**<br>CCAAGAATAGTTTTTCATGGTAC<br>sequence remains out of frame	**wild type**<br>CCTAATTTGCCTCACGAAATCAA<br>sequence remains out of frame	+

NbUGT73Q2

Plant line	sgRNA7<br>CCTCTC-AACGCACCTAAATTCTC	sgRNA2<br>GTTGACGTTGCAGCC-AAGCTGGG	T-DNA
0720-06-01	**homozygous**<br>CCTCTCAAACGCACCTAAATTCTC<br>frameshift from aa42 and stop codon at aa49	**homozygous**<br>GTTGACGTTGCAGCCAAAGCTGGG<br>sequence remains out of frame	-
0720-06-03	**homozygous**<br>CCTCTCAAACGCACCTAAATTCTC<br>frameshift from aa42 and stop codon at aa49	**homozygous**<br>GTTGACGTTGCAGCCAAAGCTGGG<br>sequence remains out of frame	+

Supplementary Figure 3. Cas9-mediated mutagenesis of four Group D UGTs (a) Schematic showing construct design (b) locations of sgRNAs (c) sequences of targets and genotypes of T1 plants

a

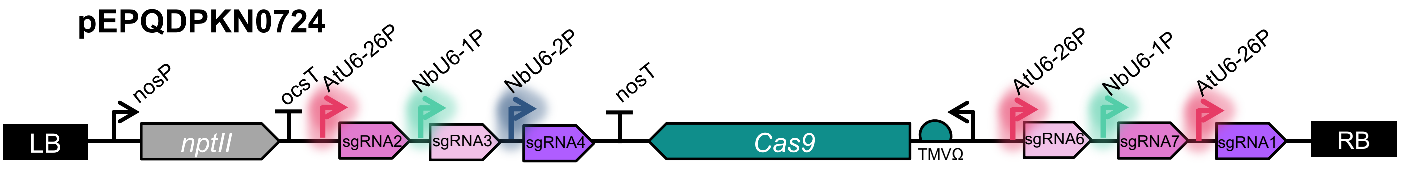

b

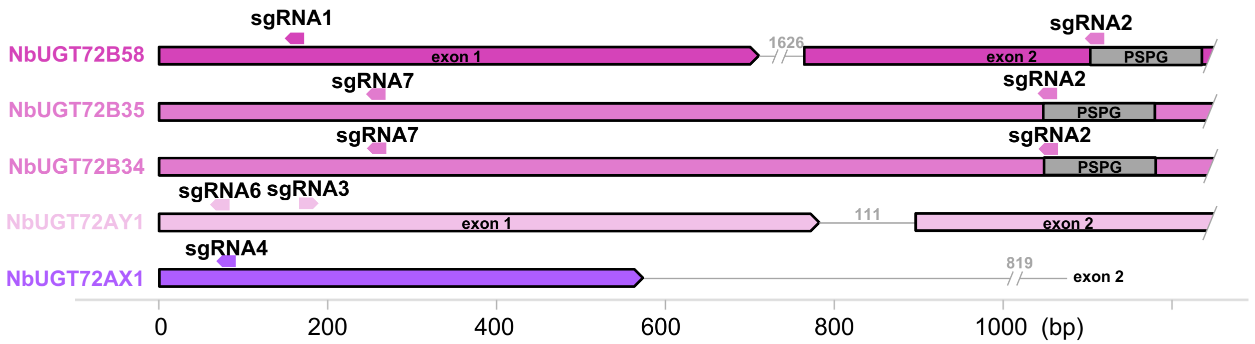

c

NbUGT72B58

Plant line	sgRNA1<br>CCTACT-AACGGTCCCATCTCTAA	sgRNA2<br>CCTAATTGGGCCCCACAAGCCCAAATC	T-DNA
0724-37-06	**homozygous**<br>CCTACTAAACGGTCCCATCTCTAA<br>frameshift at aa52 and stop codon at aa57	**wild type**<br>CCTAATTGGGCCCCACAAGCCCAAATC<br>n/a	-
0724-22a-06	**homozygous**<br>CCTACT----GGTCCCATCTCTAA<br>3 bp in-frame deletion of aa52 (not considered a loss-of-function mutation)	**wild type**<br>CCTAATTGGGCCCCACAAGCCCAAATC<br>n/a	+

NbUGT72B35

Plant line	sgRNA7<br>CCTCTTGATGTTAAAATTGAAAC	sgRNA2<br>CCTAATTGGGCCCCACAAGCTCAAATC	T-DNA
0724-37-06	**wild type**<br>CCTCTTGATGTTAAAATTGAAAC<br>n/a	**wild type**<br>CCTAATTGGGCCCCACAAGCTCAAATT<br>n/a	-
0724-22a-06	**wild type**<br>CCTCTTGATGTTAAAATTGAAAC<br>n/a	**homozygous**<br>CCTAAT--GGCCCCACAAGCTCAAATT<br>frameshift at aa350 and stop codon at aa357	+

NbUGT72B34

Plant line	sgRNA7<br>CCTCTTGATGTTAAAATTGAAAC	sgRNA2<br>CCTAA-TTGGGCCCCACAAGCTCAAATT	T-DNA
0724-37-06	**wild type**<br>CCTCTTGATGTTAAAATTGAAAC<br>n/a	**bi-allelic**<br>CCTAATTTGGGCCCCACAAGCTCAAATT<br>CCTAA--TGGGCCCCACAAGCTCAAATT<br>framshift from aa350 and stop codon aa396	-
0724-22a-06	**homozygous**<br>CCTCTTG----------------	**homozygous**<br>-------------------------ATT	+
	812 bp deletion and frameshift introducing a stop codon		

NbUGT72AY1

Plant line	sgRNA6<br>CCCAGT-TCTAGTCTTAGGCAACC	sgRNA3<br>GAAAACCACTCTCACCAATGAGG	T-DNA
0724-37-06	**homozygous**<br>CCCAGTCTCTAGTCTTAGGCAACC<br>frameshift from aa23, stop codon at aa 34	**homozygous**<br>GAAAACCACTCTCACC-ATGAGG<br>sequence back in frame, but after stop codon	-
0724-22a-06	**homozygous**<br>CCCAGTTTCTAGTCTTAGGCAACC<br>frameshift from aa23, stop codon at aa 34	**homozygous**<br>GAAAACCACTCTCACC-ATGAGG<br>sequence back in frame, but after stop codon	+

NbUGT72AX1

Plant line	sgRNA4<br>CCCTGG-CATGGGACACATCATCC	T-DNA
0724-37-06	**homozygous**<br>CCCTGG--ATGGGACACATCATCC<br>frameshift from aa26, stop codon at aa 40	-
0724-22a-06	**homozygous**<br>CCCTGGACATGGGACACATCATCC<br>frameshift from aa26, stop codon at aa 34	+

Supplementary Figure 4. Cas9-mediated mutagenesis of five Group E UGTs (a) Schematic showing construct design (b) locations of sgRNAs (c) sequences of targets and genotypes of T1 plants

a

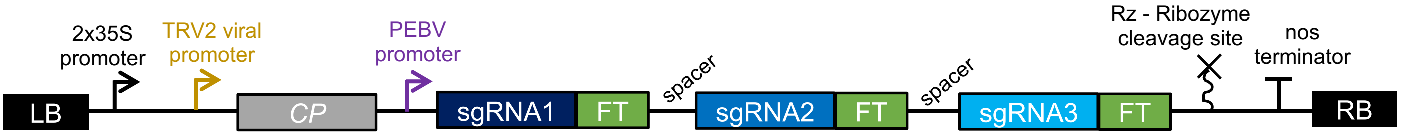

b

pEPQDKN0777

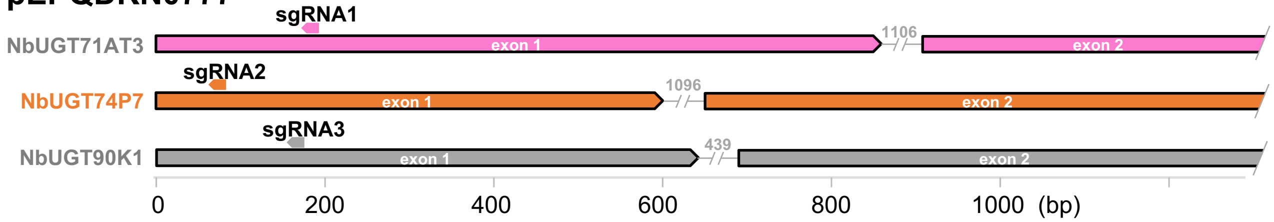

	NbUGT71AT3	NbUGT74P7	NbUGT90K1
	sgRNA1	sgRNA2	sgRNA3
	CCCTTAGACTATGGTGTTCAAAA	CCCCGCACAAGGCCACATAAATC	CCTCGCGGCCCTTTTGCGCCACC
NbCas9-0777B-01	wild type<br>CCCTTAGACTATGGTGTTCAAAA<br>n/a	homozygous<br>CCCCGC-----GCCACATAAATC<br>frameshift from aa18, stop codon at aa35	wild type<br>CCTCGCGGCCCTTTTGCGCCACC<br>n/a

pEPQDKN0778

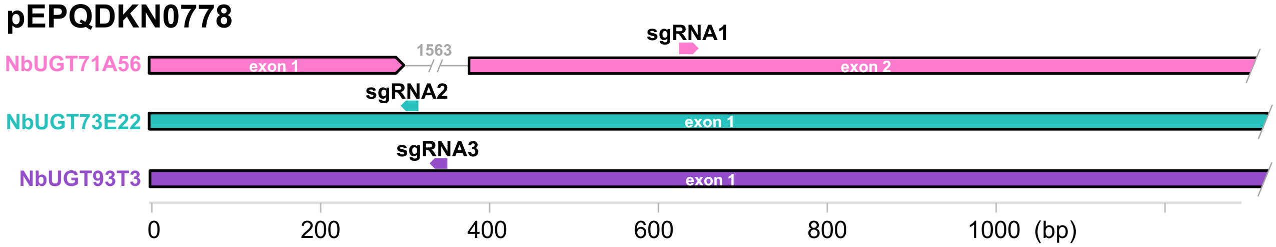

	NbUGT71A56	NbUGT73E22	NbUGT93T3
	sgRNA1	sgRNA2	sgRNA3
	TCACCTTGTACCCACT-GTGGAGG	CCCTA-TGATTGACACTGCGCGAT	CCTCTT-ATGGTCTTCCTGTTTAC
NbCas9-0778B-05	homozygous<br>TCACCTTGTACCCACTGGTGGAGG<br>frameshift from aa21, stop codon at aa54	homozygous<br>CCCTATTGATTGACACTGCGCGAT<br>frameshift from aa24, stop codon at aa26	homozygous<br>CCTCTTAATGGTCTTCCTGTTTAC<br>frameshift from aa47, stop codon at aa47

pEPQDKN0779

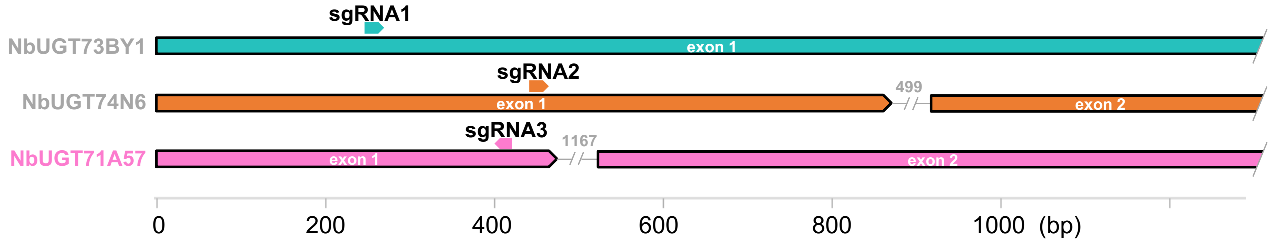

	NbUGT73BY1	NbUGT74N6	NbUGT71A57
	sgRNA1	sgRNA2	sgRNA3
	CCACACCTCTCAATGCCCAAAGG	CTACCAAGCGGAAAGCTTCGCGG	CCGCGTTTATTGCCGGTCATAAG
NbCas9-0779A-01	wild type<br>CCACACCTCTCAATGCCCAAAGG<br>n/a	wild type<br>CTACCAAGCGGAAAGCTTCGCGG<br>n/a	homozygous<br>CCGCG-TTATTGCCGGTCATAAG<br>frameshift from aa89, stop codon at aa113

Supplementary Figure 5. Cas9-mediated mutagenesis of 16 UGTs using mobile sgRNAs (a) Schematic showing construct design (b) locations of mobile sgRNAs on target genes and genotypes of target genes in E1 plants

pEPQDKN0780

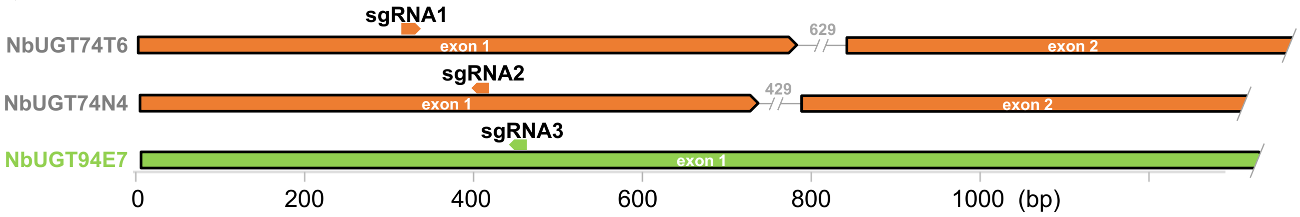

	NbUGT74T6	NbUGT74N4	NbUGT94E7
	**sgRNA1**	**sgRNA2**	**sgRNA3**
	GTATCGATACCATTTCCGATGGG	CCCTGTGAATTGCATAGTTTATG	CCCCAT-CTCAATCACGCCCTTAG
**NbCas9-0780B-01**	**wild type**<br>GTATCGATACCATTTCCGATGGG<br>n/a	**wild type**<br>CCCTGTGAATTGCATAGTTTATG<br>n/a	**homozygous**<br>CCCCATCCTCAATCACGCCCTTAG<br>frameshift from aa95, stop codon at aa100

pEPQDKN0781

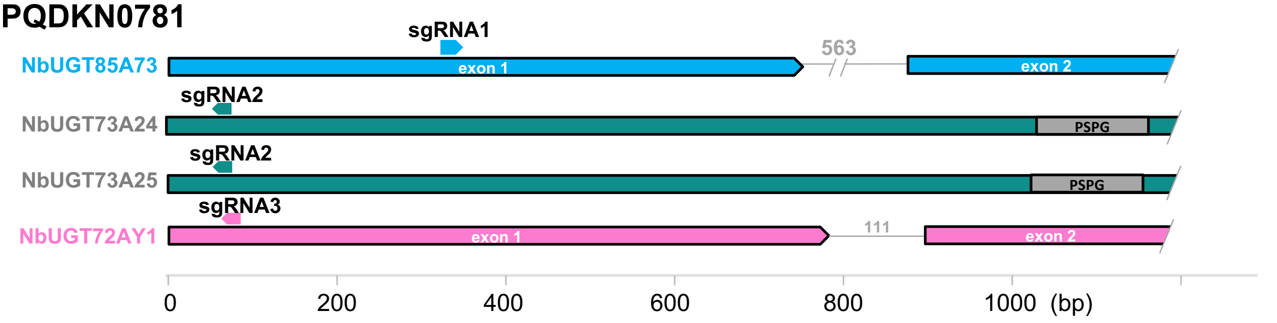

	NbUGT85A73	NbUGT73A24 and NbUGT73A25	NbUGT72AY1
	**sgRNA1**	**sgRNA2**	**sgRNA3**
	CGTGGCCCTGATTCTCTCAAGGG	CCTACACTAGACATGGCGAAGCT	CCCAGTTCTAGTCTTAGGCAACC
**NbCas9-0781B-03**	**bi-allelic**<br>CGTGGCCCTGATTCTCT-AAGGG<br>CGTGGCCCTGAT----TCAAGGG<br>frameshift at aa62/63 and stop codon at aa129	**wild type**<br>CCTACACTAGACATGGCGAAGCT<br>n/a	**homozygous**<br>CCCAG-TCTAGTCTTAGGCAACC<br>frameshift from aa23, stop codon at aa23

Supplementary Figure 5 continued. Cas9-mediated mutagenesis of 16 UGTs using mobile sgRNAs (a) Schematic showing construct design (b) locations of mobile sgRNAs on target genes and genotypes of target genes in E1 plants

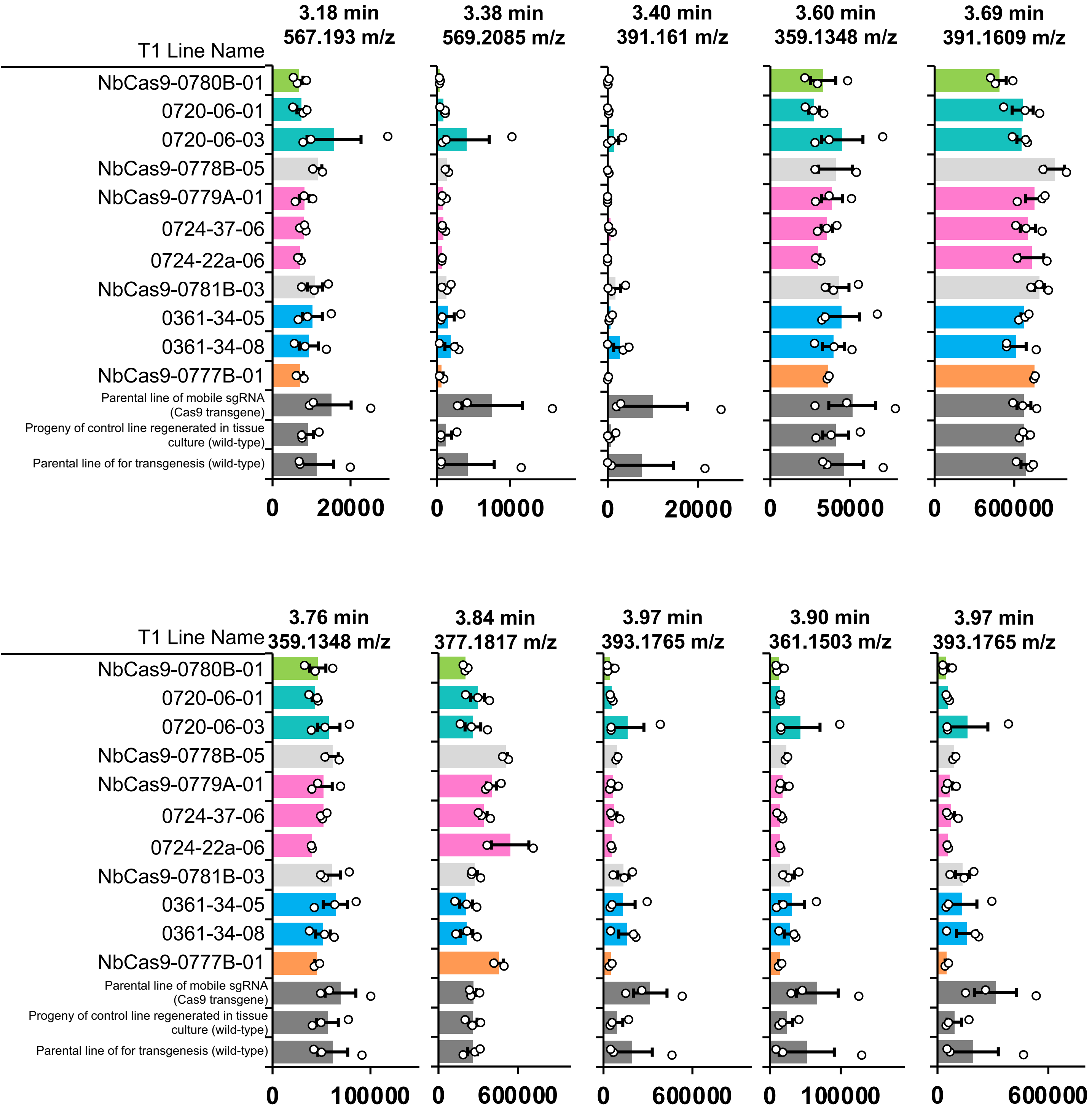

Supplementary Figure 6. Abundance of derivative peaks in lines of *N. benthamiana* infiltrated with the geraniol biosynthesis pathway (P19 + DXS + GPPS + GES). Values and error bars represent the mean and the standard error of n=3 biological replicates (independent leaf samples). For the pairwise comparison of all mutated lines to all three control lines (gray bars), means followed by a common Greek letter (α,β,γ,δ) are not significantly different by a one-way ANOVA with post-hoc Tukey HSD at the 5% level of significance. Plots with no Greek letter values have no experimental lines with significant differences from the three control lines.

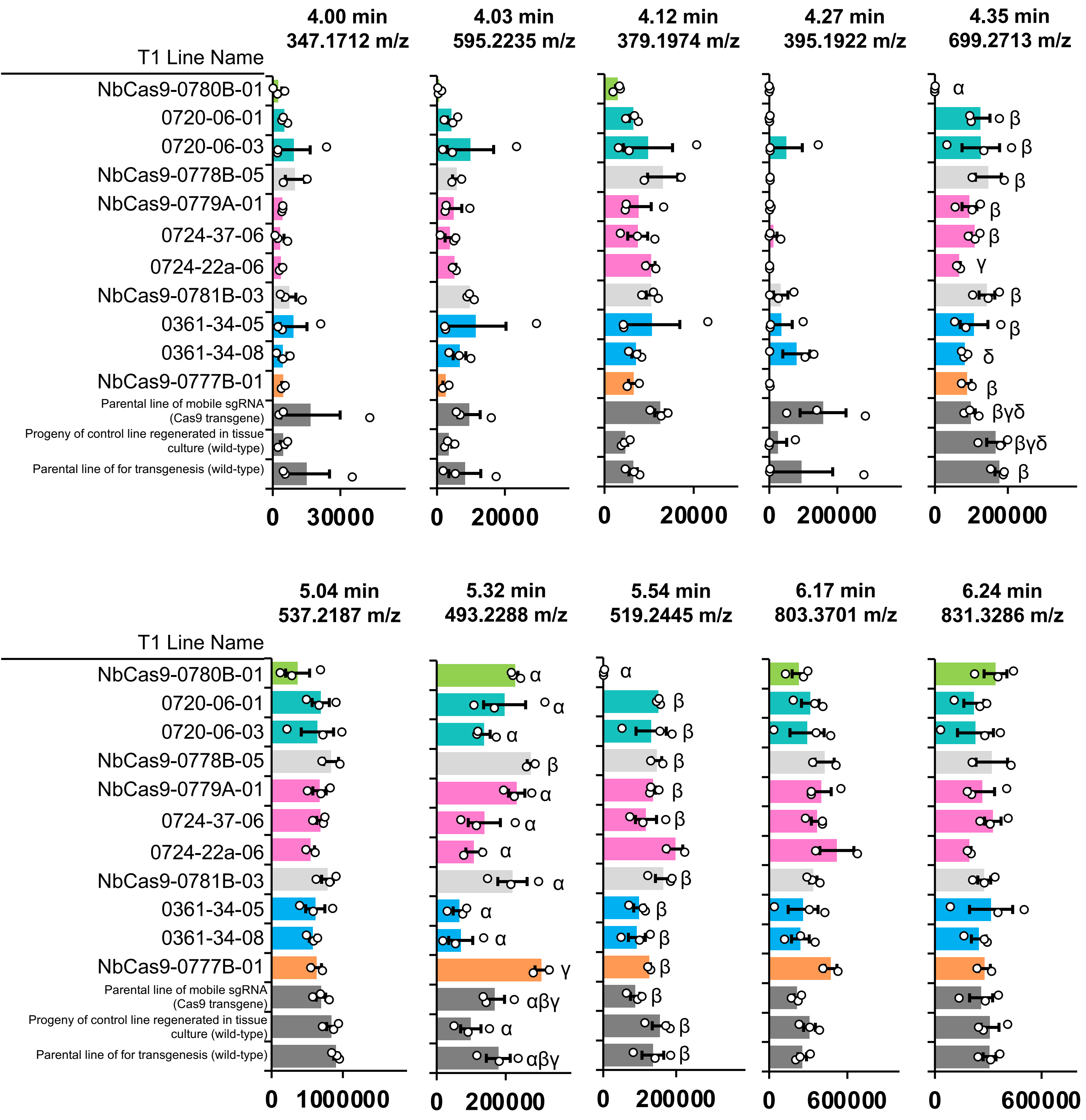

Supplementary Figure 6 continued. Abundance of derivative peaks in lines of *N. benthamiana* infiltrated with the geraniol biosynthesis pathway (P19 + DXS + GPPS + GES). Values and error bars represent the mean and the standard error of n=3 biological replicates (independent leaf samples). For the pairwise comparison of all mutated lines to all three control lines (gray bars), means followed by a common Greek letter (α,β,γ,δ) are not significantly different by a one-way ANOVA with post-hoc Tukey HSD at the 5% level of significance. Plots with no Greek letter values have no experimental lines with significant differences from the three control lines.

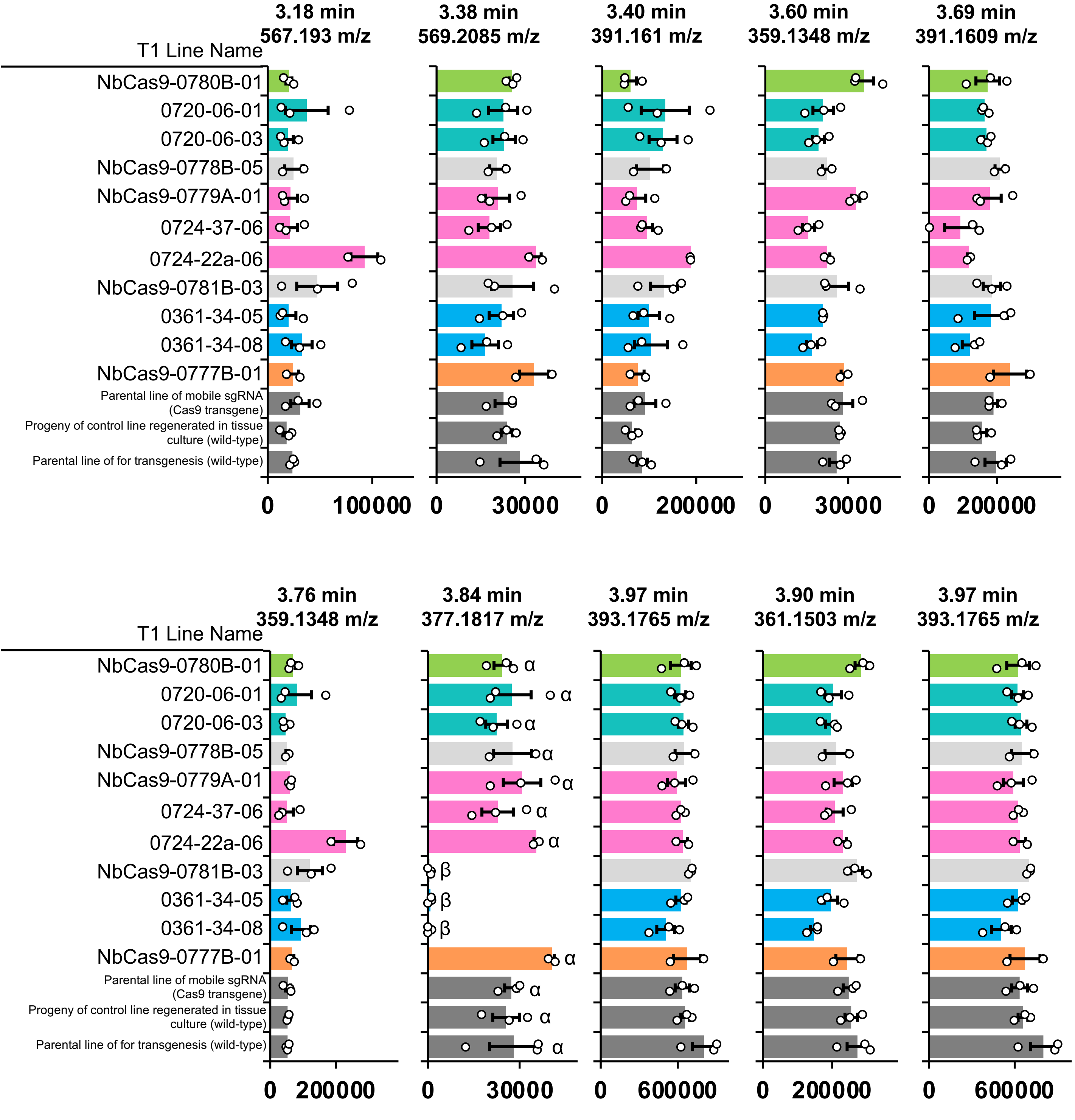

Supplementary Figure 7. Abundance of derivative peaks in lines of *N. benthamiana* infiltrated with the nepetalactol biosynthesis pathway (P19 + DXS + GPPS + GES + G8H + GOR + ISY + MLPL). Values and error bars represent the mean and the standard error of n=3 biological replicates (independent leaf samples). For the pairwise comparison of all mutated lines to all three control lines (gray bars), means followed by a common Greek letter (α,β,γ,δ) are not significantly different by a one-way ANOVA with post-hoc Tukey HSD at the 5% level of significance. Plots with no Greek letter values have no experimental lines with significant differences from the three control lines.

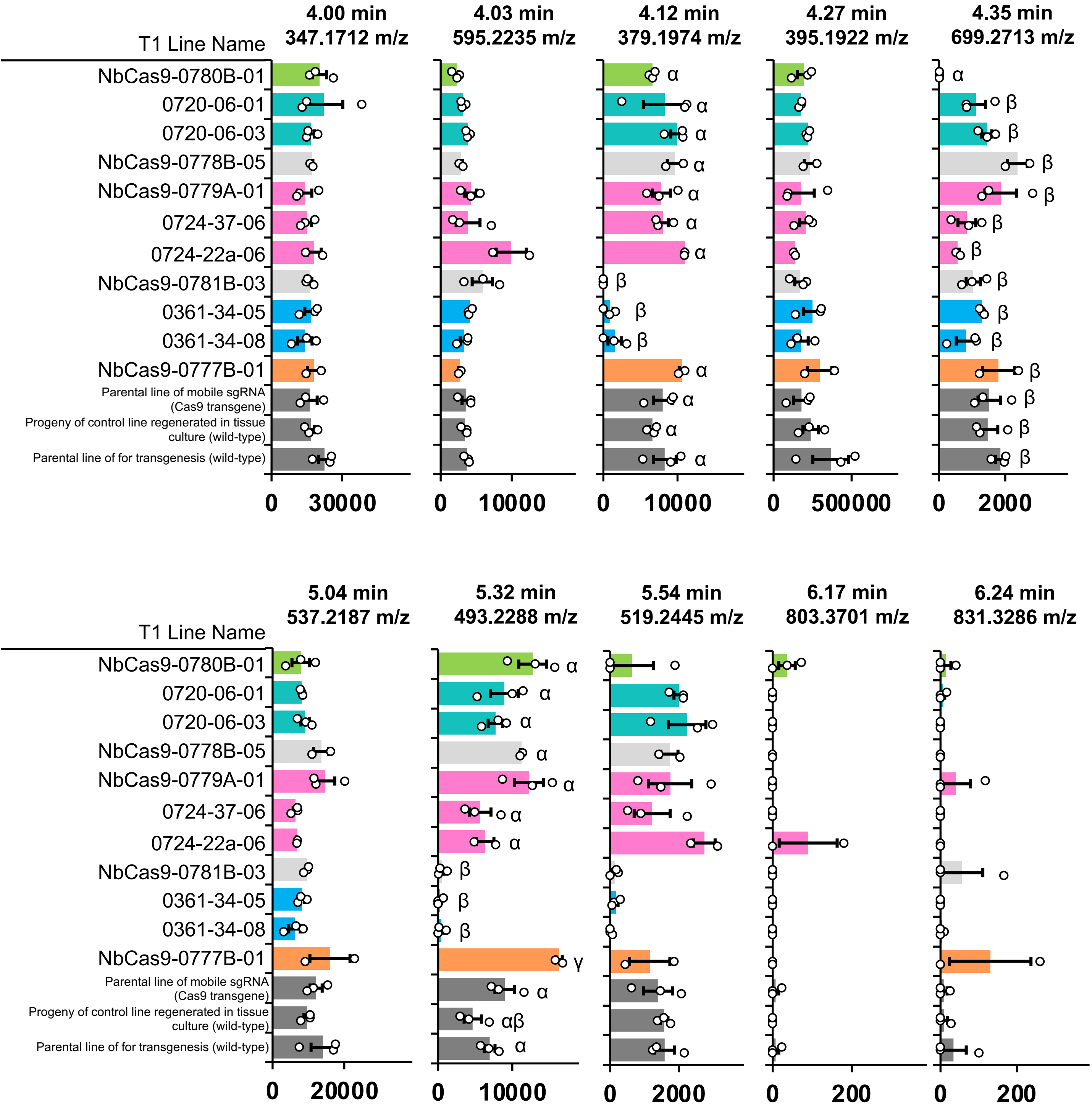

Supplementary Figure 7 continued. Abundance of derivative peaks in lines of *N. benthamiana* infiltrated with the nepetalactol biosynthesis pathway (P19 + DXS + GPPS + GES + G8H + GOR + ISY + MLPL). Values and error bars represent the mean and the standard error of n=3 biological replicates (independent leaf samples). For the pairwise comparison of all mutated lines to all three control lines (gray bars), means followed by a common Greek letter (α,β,γ,δ) are not significantly different by a one-way ANOVA with post-hoc Tukey HSD at the 5% level of significance. Plots with no Greek letter values have no experimental lines with significant differences from the three control lines.

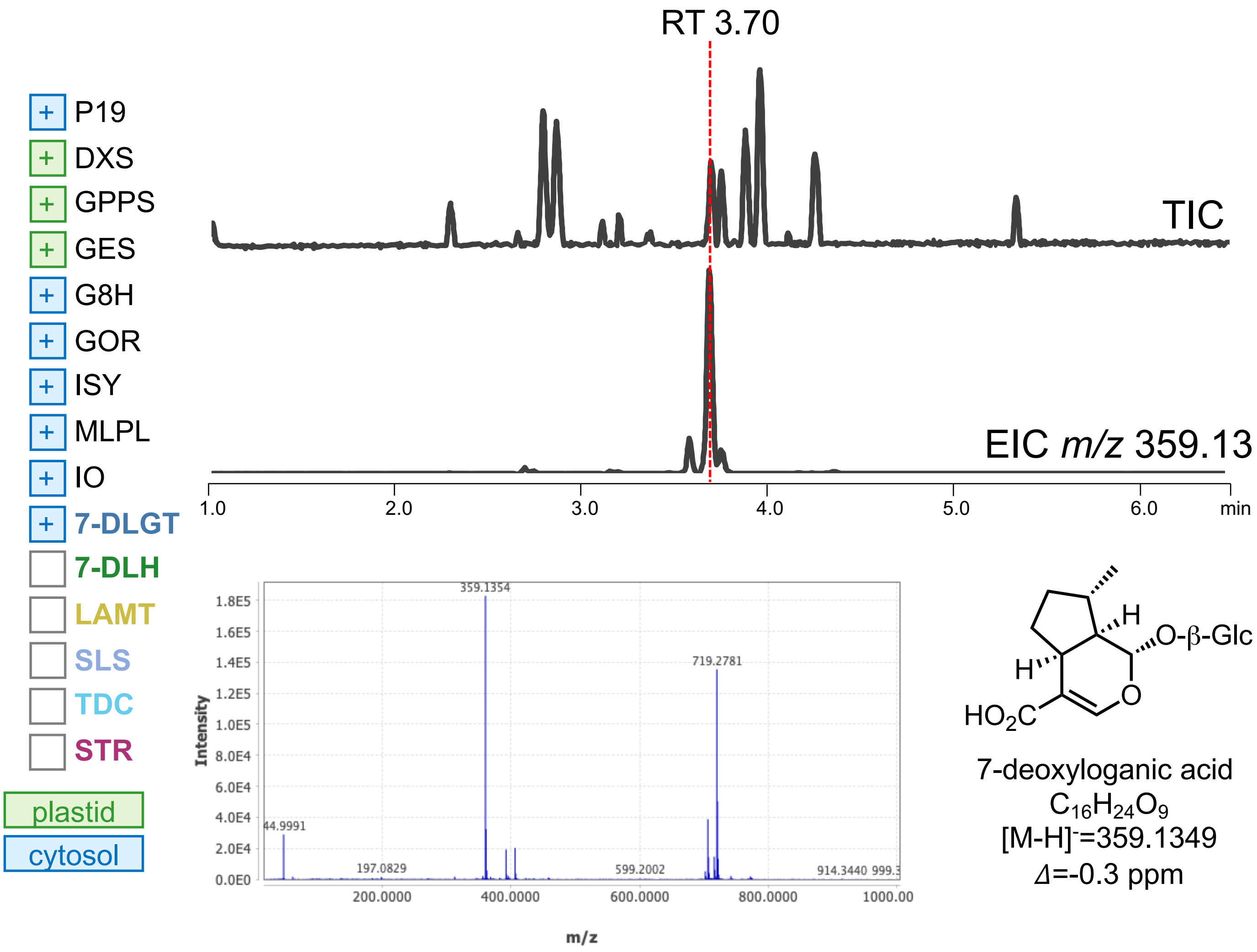

Supplementary Figure 8. Detection of 7-deoxyloganic acid following transient expression of pathway genes in *N. benthamiana*

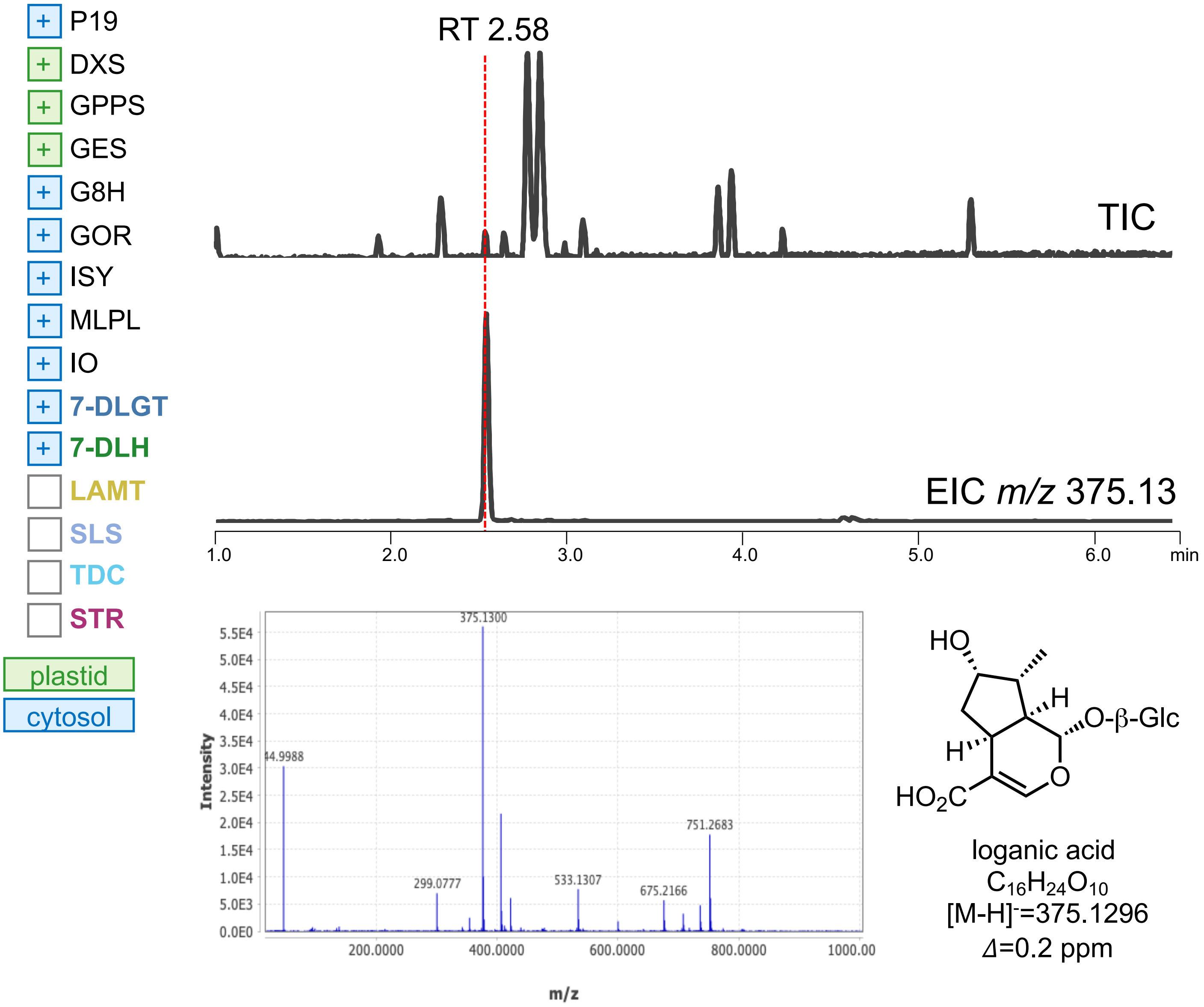

Supplementary Figure 9. Detection of loganic acid following transient expression of pathway genes in *N. benthamiana*

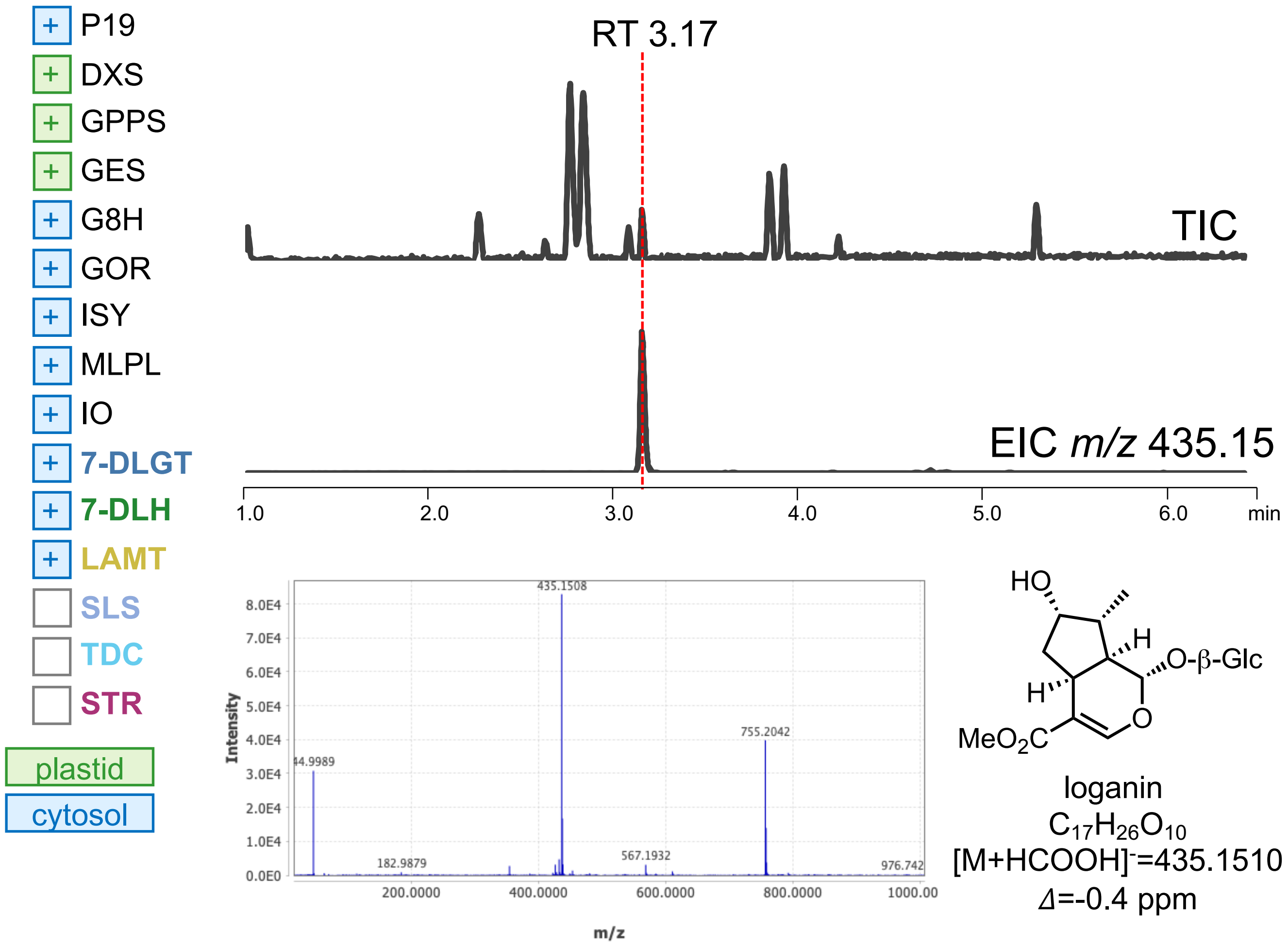

Supplementary Figure 10. Detection of loganin following transient expression of pathway genes in *N. benthamiana*

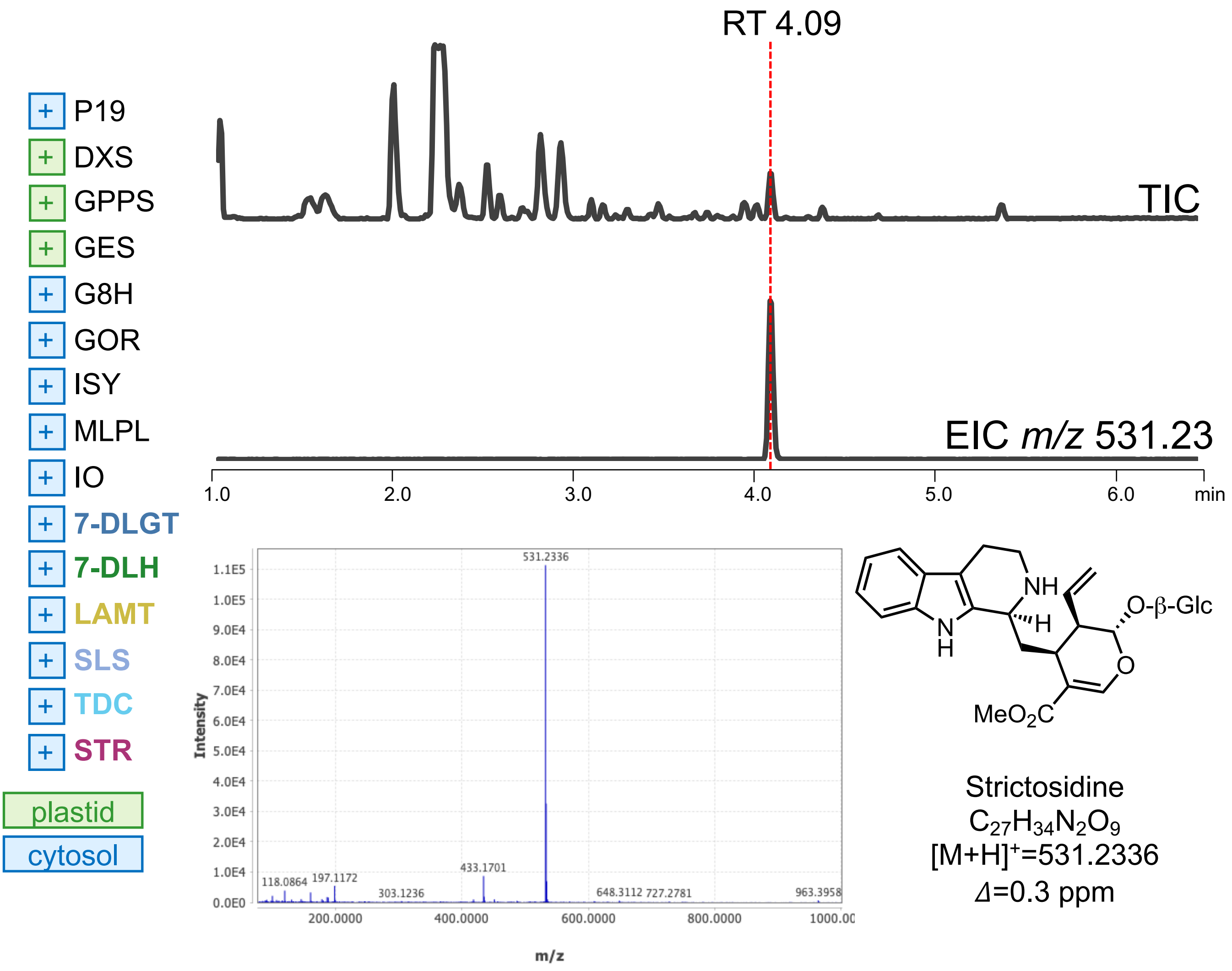

Supplementary Figure 11. Detection of strictosidine following transient following transient expression of pathway genes in *N. benthamiana*

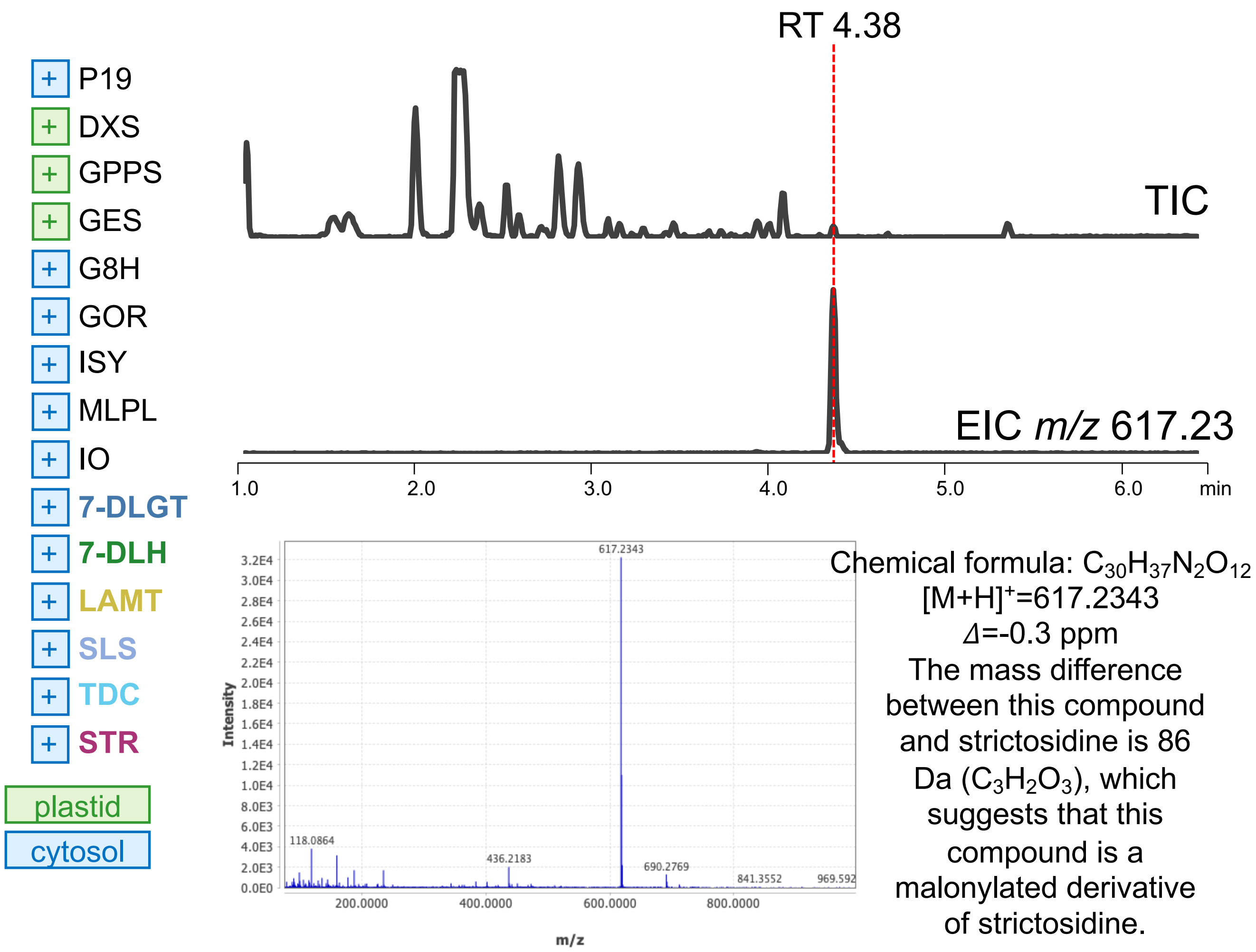

Supplementary Figure 12. Detection of putative malonyl-strictosidine following transient expression of pathway genes in *N. benthamiana*

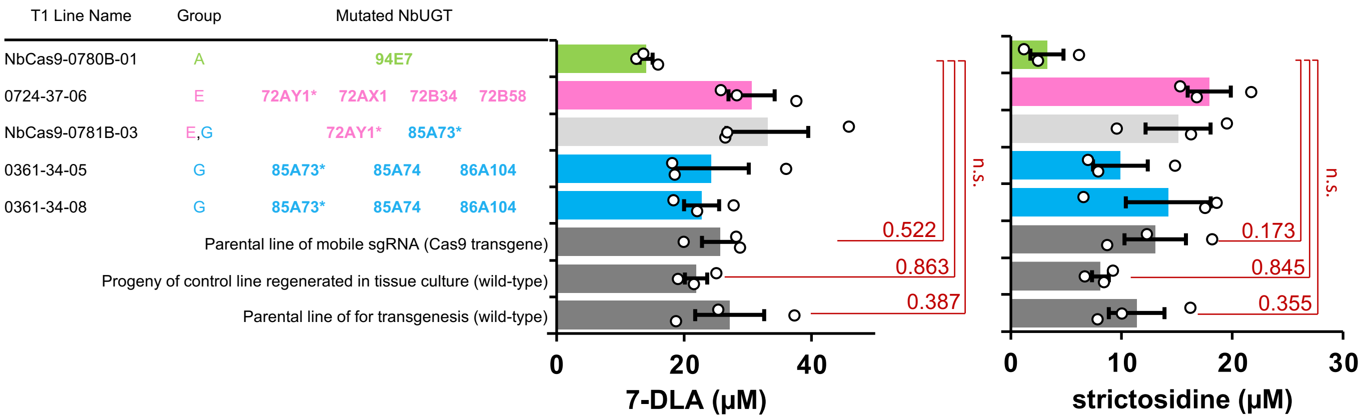

Supplementary Figure 13. Quantification of strictosidine following transient expression of pathway genes in mutated and wild-type *N. benthamiana* by UPLC/MS analysis. Values and error bars represent the mean and the standard error of n=3 biological replicates (independent leaf samples). Red numerals indicate exact p-value of a one-way ANOVA with post-hoc Tukey HSD test. n.s. = not significant; p-value > 0.05.

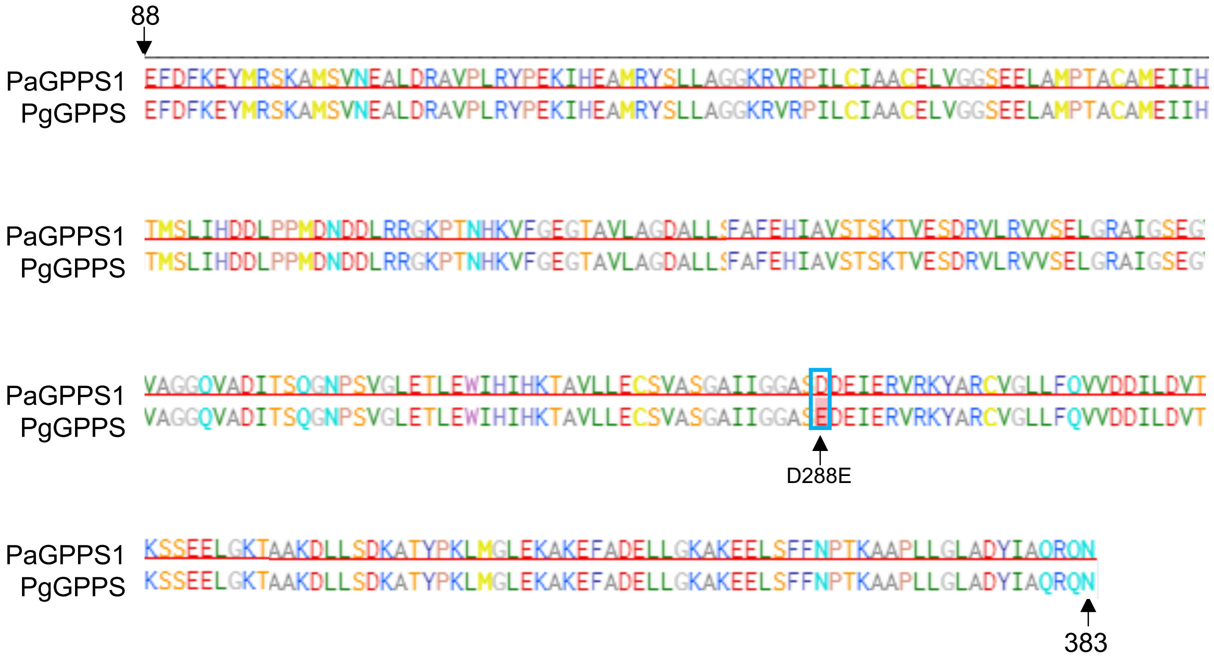

Supplementary Figure 14. Amino acid sequence alignment of GPPS enzymes. PaGPPS1 (GQ369788.1) - Used in this study and in Miettinen *et al. Nat. Comm.* 5:3606 (2014) and Dong *et al. New Phytologist* 209:2 679-690 (2016). PgGPPS (AHE15048.1) shown by Dudley *et al. Metab. Eng.* 61 251-260 (2020) to produce higher levels of the monoterpene limonene relative to six other GPPS sequences commonly used in terpene metabolic engineering.

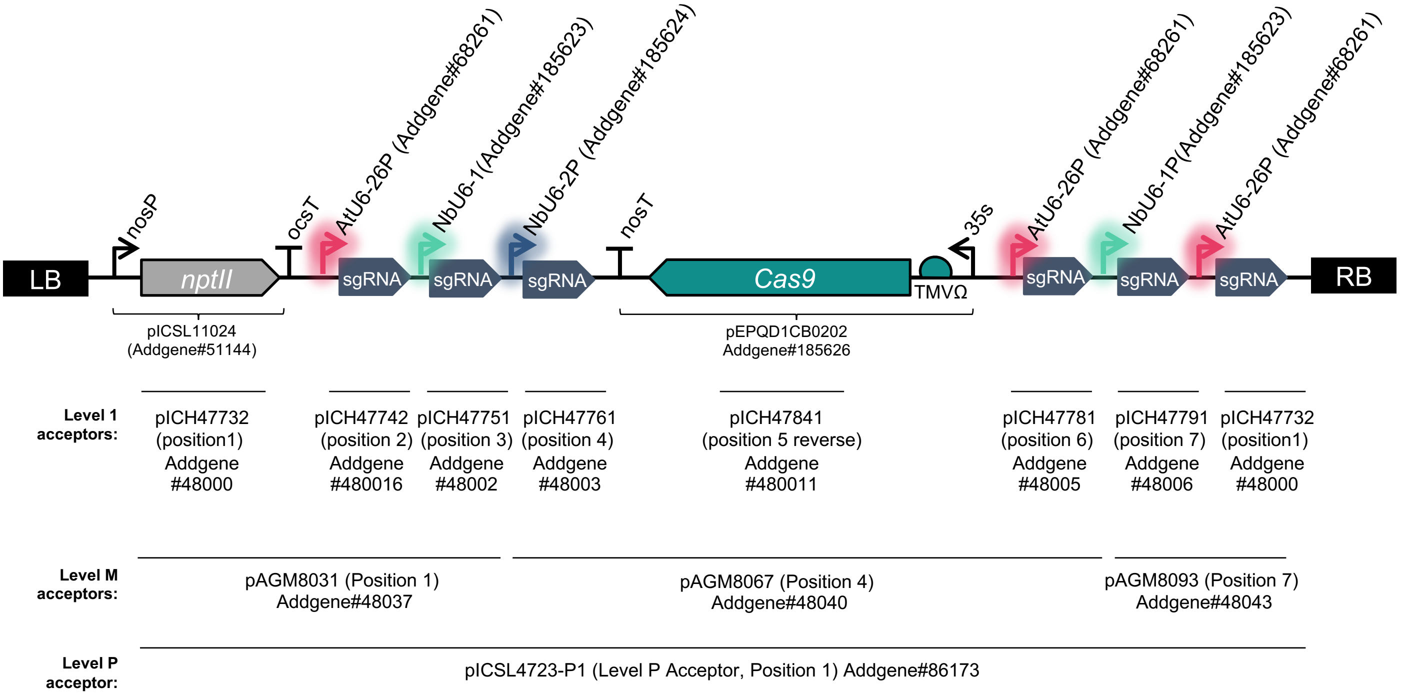

Supplementary Figure 15. Design and hierarchical assembly of binary constructs used for Cas9-mediated mutagenesis of *Nicotiana benthamiana.*

Supplementary Table 1 LC/MS analysis and putative identification of iridoid pathway derivatives produced by pathway expression in *N. benthamiana*.

Peak number	RT (min)	m/z	Putative annotation	Ion Formula	M-H	M-H2O-H	M+HCOOH-H	P19 + **GPPS + GES**	P19 + **DXS + GPPS + GES**	P19 + **DXS + GPPS + GES + G8H**	P19 + **DXS + GPPS + GES + G8H + GOR**	P19 + **DXS + GPPS + GES + G8H + GOR + ISY**	P19 + **DXS + GPPS + GES + G8H + GOR + ISY + MLPL**	m/z
-	3.18	567.1930	Dihexosyl hydroxycarboxy geranial (+HCOOH-H)	C23H35O16	C23H36O16	C23H38O17	C22H34O14				y		y	567.1930
-	3.38	569.2085	Dihexosyl hydroxycarboxy geraniol (+HCOOH-H)	C23H37O16	C23H38O16	C23H40O17	C22H36O14				y	y	y	569.2085
-	3.40	391.1610	Hexosyl carboxygeranial (+HCOOH-H)	C17H27O10	C17H28O10	C17H30O11	C16H26O8						y	391.1610
-	3.60	359.1348	Pentosyl dihydroxy geranial (+HCOOH-H) or hexosyl carboxygeranic acid	C16H23O9	C16H24O9	C16H26O10	C15H22O7	y	y	y	y	y		359.1348
-	3.69	391.1609	Hexosyl dihydroxy geranial (+HCOOH-H)	C17H27O10	C17H28O10	C17H30O11	C16H26O8	y	y	y	y	y	y	391.1609
-	3.76	359.1348	Pentosyl dihydroxy geranial (+HCOOH-H)	C16H23O9	C16H24O9	C16H26O10	C15H22O7	y	y	y	y			359.1348
1	3.84	377.1817	Hexosyl hydroxygeraniol (+HCOOH-H)	C17H29O9	C17H30O9	C17H32O10	C16H28O7	y	y	y	y			377.1817
-	3.97	393.1765	Hexosyl dihydroxygeraniol (+HCOOH-H)	C17H29O10	C17H30O10	C17H32O11	C16H28O8	y	y	y	y			393.1765
-	3.90	361.1503	Pentosyl hydroxygeranic acid (+HCOOH-H)	C16H25O9	C16H26O9	C16H28O10	C15H24O7	y	y	y	y	y	y	361.1503
-	3.97	393.1765	Hexosyl carboxygeraniol (+HCOOH-H)	C17H29O10	C17H30O10	C17H32O11	C16H28O8		y	y	y	y	y	393.1765
-	4.00	347.1712	Pentosyl hydroxygeraniol (+HCOOH-H)	C16H27O8	C16H28O8	C16H30O9	C15H26O6			y				347.1712
-	4.03	595.2235	Malonyl hexosyl pentosyl hydroxygeraniol (+HCOOH-H)	C25H39O16	C25H40O16	C25H42O17	C24H38O14			y				595.2235
2	4.12	379.1974	Hexosyl hydroxycitronellal (+HCOOH-H)	C17H31O9	C17H32O9	C17H34O10	C16H30O7			y	y			379.1974
-	4.27	395.1922	Hexosyl dihydroxycitronellol (+HCOOH-H)	C17H31O10	C17H32O10	C17H34O11	C16H30O8		y	y	y	y	y	395.1922
3	4.35	699.2713	Trihexosyl geranic acid (+HCOOH-H)	C29H47O19	C29H48O19	C29H50O20	C28H46O17		y	y	y			699.2713
-	5.04	537.2187	Dihexosyl geranic acid (+HCOOH-H)	C23H37O14	C23H38O14	C23H40O15	C22H36O12	y	y					537.2187
4	5.32	493.2288	Pentosyl hexosyl geraniol (+HCOOH-H)	C22H37O12	C22H38O12	C22H40O13	C21H36O10	y	y					493.2288
5	5.54	519.2445	Acetyl dihexosyl geraniol	C24H39O12	C24H40O12	C24H42O13	C23H38O10	y	y					519.2445
-	6.17	803.3701	Malonyl hexosyl geraniol (dimer)	C38H59O18	C38H60O18	C38H62O19	C37H58O16	y	y					803.3701
-	6.24	831.3286	Malonyl hexosyl geranic acid (dimer)	C38H55O20	C38H56O20	C38H58O21	C37H54O18	y	y					831.3286

Supplementary Table 2 Expression levels and protein features of *N. benthamiana* UDP-glycosyltransferases (UGTs) Relative activity on geraniol is reported from Sun et al. 2019[1]

UGT	Group code	Mutagenesis Plasmid	Status in T0 plants	Geraniol activity (relative)	Length (amio acids)	His	Asp	PSPG	GSS	GFP/ Untransformed			Low geraniol /GFP			High geraniol /GFP			Nepetalactol /GFP			DESeq2 Normalized counts				
										log2 fold change	standard error	adjusted p-value	log2 fold change	standard error	adjusted p-value	log2 fold change	standard error	adjusted p-value	log2 fold change	standard error	adjusted p-value	untrans-formed	GFP	Low geraniol	High geraniol	Nepeta-lactol
NbUGT90K1	Group C	pEPQDKN0777	targeted		468	Y	Y	Y	Y	5.180	0.169	7.53E-204	0.109	0.161	8.76E-01	-0.378	0.161	1.40E-01	-0.202	0.161	3.83E-01	234	9698	8983	6890	7796
NbUGT71AT3	Group E	pEPQDKN0777	targeted		476	Y	Y	Y	Y	8.660	0.228	0.00E+00	0.084	0.182	9.30E-01	0.100	0.182	8.48E-01	0.792	0.182	1.11E-03	28	15346	14462	15514	25270
NbUGT74P7	Group L	pEPQDKN0777	mutated		454	Y	Y	Y	Y	4.827	0.164	1.38E-187	-0.034	0.158	9.74E-01	0.292	0.157	2.96E-01	0.224	0.157	3.13E-01	292	8520	8724	10707	10209
NbUGT73E22	Group D	pEPQDKN0778	mutated		488	Y	Y	Y	Y	-1.839	0.378	2.95E-06	0.388	0.380	7.58E-01	1.862	0.378	5.00E-05	2.044	0.378	4.24E-05	602	202	151	613	702
NbUGT71A56	Group E	pEPQDKN0778	mutated		399	Y	Y	Y	no	-1.267	0.537	2.88E-02	0.347	0.537	8.84E-01	1.330	0.537	1.10E-01	2.046	0.537	4.36E-03	2331	1069	792	2458	4448
NbUGT93T3	Group O	pEPQDKN0778	mutated		473	Y	Y	Y	Y	-1.998	0.128	8.99E-54	0.127	0.132	7.79E-01	1.135	0.129	6.80E-16	-0.070	0.132	7.50E-01	1668	451	413	913	393
NbUGT73BY1	Group D	pEPQDKN0779	targeted		493	Y	Y	Y	Y	0.444	0.513	4.56E-01	0.081	0.512	9.82E-01	0.430	0.512	7.33E-01	2.494	0.511	2.50E-04	174	270	252	360	1912
NbUGT71A57	Group E	pEPQDKN0779	mutated		484	Y	Y	Y	Y	-0.485	0.579	4.71E-01	-0.292	0.581	9.20E-01	0.185	0.580	9.21E-01	3.285	0.576	1.28E-05	59	29	38	45	635
NbUGT74N6	Group L	pEPQDKN0779	targeted		458	Y	Y	Y	Y	2.582	0.560	1.01E-05	0.024	0.551	9.96E-01	1.104	0.550	2.39E-01	2.956	0.549	4.65E-05	11	134	131	335	1527
NbUGT94E7	Group A	pEPQDKN0780	mutated		327	Y	Y	Y	no	4.769	0.478	1.74E-22	-0.119	0.473	9.68E-01	0.880	0.473	2.93E-01	2.262	0.473	3.10E-04	38	1902	2090	4188	12300
NbUGT74N4	Group L	pEPQDKN0780	targeted		461	Y	Y	Y	Y	3.328	0.434	8.83E-14	-0.146	0.433	9.53E-01	0.691	0.433	4.03E-01	2.085	0.433	2.90E-04	403	4980	5578	9515	27698
NbUGT74T6	Group L	pEPQDKN0780	targeted		457	Y	Y	Y	Y	1.421	0.226	1.29E-09	-0.299	0.226	6.34E-01	-0.113	0.226	8.64E-01	0.986	0.226	1.06E-03	8910	19815	24513	22619	49467
NbUGT85A73*	Group G	pEPQDPKN0361	mutated	100	486	Y	Y	Y	Y	-1.293	0.166	2.87E-14	0.012	0.166	9.93E-01	0.149	0.166	7.08E-01	0.605	0.166	6.28E-03	7224	2934	2910	3231	4453
NbUGT85A104	Group G	pEPQDPKN0361	mutated		496	Y	Y	Y	Y	-3.836	0.301	5.56E-36	1.865	0.310	4.19E-07	0.271	0.333	7.44E-01	0.661	0.322	1.23E-01	341	83	21	26	35
NbUGT85A74	Group G	pEPQDPKN0361	mutated		462	Y	Y	Y	no	-1.946	0.168	5.54E-30	0.398	0.170	2.06E-01	0.747	0.170	4.35E-04	0.616	0.170	6.64E-03	1525	514	388	657	599
NbUGT73A24*	Group D	pEPQDPKN0720	mutated	81	477	Y	Y	Y	Y	0.671	0.267	1.92E-02	0.053	0.267	9.76E-01	1.120	0.266	9.27E-04	1.217	0.266	5.84E-04	3752	6326	6087	13633	14616
NbUGT73A25*	Group D	pEPQDPKN0720	mutated	47	477	Y	Y	Y	Y	2.688	0.344	2.79E-14	0.114	0.341	9.54E-01	1.114	0.341	1.85E-02	1.614	0.341	3.65E-04	239	1929	1773	4038	5836
NbUGT73A32	Group D	pEPQDPKN0720	mutated		412	Y	Y	Y	no	1.309	0.131	1.58E-22	-0.167	0.125	6.30E-01	0.117	0.125	6.91E-01	-0.181	0.125	3.03E-01	271	602	677	735	596
NbUGT73Q2	Group D	pEPQDPKN0720	mutated		486	Y	no	Y	Y	5.389	0.356	1.47E-50	0.037	0.345	9.90E-01	0.138	0.345	8.99E-01	1.671	0.345	2.77E-04	55	3321	3231	3578	11082
NbUGT72AX1	Group E	pEPQDPKN0724	mutated	2	489	Y	Y	Y	Y	-0.502	0.233	4.73E-02	-0.177	0.233	8.51E-01	-0.427	0.233	3.05E-01	-0.740	0.233	1.69E-02	11298	6959	7898	5824	4658
NbUGT72AY1*	Group E	pEPQDPKN0724	mutated	23	478	Y	Y	Y	Y	-0.549	0.366	1.78E-01	-0.647	0.370	4.46E-01	-0.379	0.369	6.55E-01	-0.768	0.370	1.18E-01	226	92	150	113	84
NbUGT72B34	Group E	pEPQDPKN0724	mutated	11	479	Y	Y	Y	Y	1.979	0.125	5.89E-55	-0.097	0.123	8.43E-01	-0.070	0.123	8.38E-01	0.356	0.123	2.77E-02	1028	3831	4099	3902	5258

NbUGT72B35	Group E	pEPQDPKN0724 mutated	1	479	Y	Y	Y	Y	-0.120	0.118	3.73E-01	0.042	0.118	9.50E-01	0.575	0.117	5.76E-05	0.334	0.117	3.08E-02	4025	3811	3701	5530	4673
NbUGT72B58	Group E	pEPQDPKN0724 mutated		479	Y	Y	Y	Y	-1.049	0.123	9.74E-17	-0.084	0.126	8.80E-01	0.244	0.125	2.60E-01	-0.311	0.126	6.02E-02	1158	525	557	660	448
NbUGT79A16	Group A	*not targeted*		472	Y	Y	Y	no	0.579	0.245	2.88E-02	-0.122	0.243	9.20E-01	-0.135	0.243	8.44E-01	-0.360	0.244	2.91E-01	201	279	305	277	235
NbUGT91A20	Group A	*not targeted*		490	Y	Y	Y	no	-1.337	0.250	2.71E-07	-0.450	0.256	4.41E-01	-0.968	0.260	4.83E-03	-0.848	0.258	1.36E-02	527	146	202	101	110
NbUGT91R10	Group A	*not targeted*		467	Y	Y	Y	no	-1.955	0.259	2.19E-13	0.894	0.262	2.05E-02	0.939	0.263	7.58E-03	-0.576	0.273	1.11E-01	362	169	89	175	58
NbUGT91S4	Group A	*not targeted*		473	Y	Y	Y	no	3.781	0.175	1.87E-101	-0.544	0.137	3.99E-03	-0.643	0.138	1.55E-04	0.416	0.135	1.97E-02	50	494	722	461	967
NbUGT94AQ1	Group A	*not targeted*		455	Y	Y	Y	no	0.678	0.433	1.58E-01	-0.039	0.433	9.91E-01	0.992	0.432	1.52E-01	2.085	0.432	2.85E-04	710	1171	1207	2605	6034
NbUGT94AR1	Group A	*not targeted*		467	Y	Y	Y	Y	1.469	0.189	4.35E-14	0.082	0.177	9.29E-01	-0.267	0.179	4.49E-01	-0.273	0.178	2.71E-01	101	304	287	238	236
NbUGT94U2	Group A	*not targeted*		444	Y	Y	Y	no	-0.721	0.629	3.13E-01	0.140	0.631	9.72E-01	0.636	0.630	6.63E-01	3.505	0.626	2.01E-05	36	20	17	34	444
NbUGT89D20	Group B	*not targeted*		468	Y	Y	Y	Y	-0.994	0.252	1.68E-04	-0.004	0.253	9.98E-01	0.390	0.252	4.26E-01	0.995	0.251	3.08E-03	949	464	465	615	950
NbUGT89V1	Group B	*not targeted*		482	Y	Y	Y	Y	2.554	0.101	3.43E-138	-0.229	0.091	1.52E-01	-0.030	0.091	9.19E-01	0.010	0.090	9.54E-01	284	1440	1689	1654	1701
NbUGT90A22	Group C	*not targeted*		465	Y	Y	Y	Y	-1.188	0.181	2.10E-10	-0.016	0.182	9.91E-01	0.045	0.182	9.43E-01	-0.524	0.182	2.92E-02	2952	1263	1277	1318	882
NbUGT73AB10	Group D	*not targeted*		492	Y	Y	Y	Y	6.594	0.302	8.71E-104	0.175	0.182	7.79E-01	-0.184	0.182	6.63E-01	-0.181	0.182	5.07E-01	7	1154	1020	896	897
NbUGT73BZ1	Group D	*not targeted*		493	Y	Y	Y	Y	0.292	0.186	1.58E-01	0.191	0.186	7.56E-01	0.174	0.186	6.93E-01	0.047	0.186	8.88E-01	1227	1724	1507	1703	1558
NbUGT73E20	Group D	*not targeted*		497	Y	Y	Y	Y	0.070	0.254	8.24E-01	-0.179	0.254	8.68E-01	0.175	0.253	7.93E-01	1.117	0.252	8.58E-04	524	485	552	625	1229
NbUGT73E21	Group D	*not targeted*		496	Y	Y	Y	Y	0.753	0.294	1.70E-02	-0.013	0.294	9.96E-01	0.759	0.294	8.97E-02	1.549	0.294	6.74E-05	1534	2629	2654	4606	8168
NbUGT73E23	Group D	*not targeted*		495	Y	no	Y	Y	0.492	0.098	1.33E-06	0.103	0.094	7.29E-01	-0.048	0.095	8.62E-01	0.128	0.093	3.33E-01	368	556	517	500	566
NbUGT71AJ1	Group E	*not targeted*		476	Y	Y	Y	Y	1.318	0.082	3.72E-57	-0.513	0.080	5.82E-08	-0.411	0.080	2.16E-05	-0.106	0.080	3.52E-01	1675	2934	4190	3147	3890
NbUGT71AT2	Group E	*not targeted*		483	Y	Y	Y	Y	-0.247	0.044	8.20E-08	-0.078	0.045	4.47E-01	0.049	0.045	6.25E-01	-0.061	0.045	3.32E-01	6339	5059	5341	5525	5118
NbUGT71AT4	Group E	*not targeted*		481	Y	Y	Y	Y	-0.310	0.556	6.41E-01	-0.034	0.556	9.94E-01	0.199	0.556	9.11E-01	-0.144	0.556	8.84E-01	6978	5193	5346	6342	4718
NbUGT71AT5	Group E	*not targeted*		380	no	Y	Y	Y	-1.179	0.118	1.09E-22	-0.118	0.124	7.83E-01	0.272	0.122	1.69E-01	0.003	0.122	9.89E-01	643	260	282	341	283
NbUGT71AU1	Group E	*not targeted*		493	Y	Y	Y	Y	0.482	0.111	3.45E-05	0.036	0.109	9.54E-01	0.077	0.109	7.87E-01	-0.045	0.109	8.07E-01	543	779	759	801	735
NbUGT71AV1	Group E	*not targeted*		483	Y	Y	Y	Y	-0.438	0.157	8.97E-03	-0.217	0.159	6.15E-01	0.145	0.158	7.01E-01	-0.028	0.158	9.24E-01	459	290	338	374	332
NbUGT71X3	Group E	*not targeted*		359	no	no	Y	Y	1.759	0.104	2.94E-62	-0.017	0.099	9.79E-01	-0.243	0.100	1.18E-01	0.291	0.099	2.53E-02	475	1603	1622	1369	1988
NbUGT71X4	Group E	*not targeted*		482	no	Y	Y	Y	0.891	0.079	3.03E-28	-0.045	0.077	8.99E-01	-0.167	0.077	1.88E-01	0.062	0.076	6.04E-01	831	1495	1544	1374	1612
NbUGT78C2	Group F	*not targeted*		444	Y	Y	Y	Y	-1.542	0.258	7.60E-09	0.414	0.264	5.24E-01	0.858	0.262	1.77E-02	0.095	0.266	8.37E-01	206	92	68	126	72
NbUGT76A4	Group H	*not targeted*		482	Y	Y	Y	Y	0.587	0.070	3.03E-16	0.000	0.069	1.00E+00	0.121	0.069	3.33E-01	-0.045	0.069	6.85E-01	1429	2151	2150	2339	2083
NbUGT76A5	Group H	*not targeted*		482	Y	Y	Y	Y	0.442	0.155	7.50E-03	0.221	0.148	5.58E-01	0.183	0.149	5.64E-01	0.277	0.147	1.60E-01	130	207	177	202	216
NbUGT87AB1	Group J	*not targeted*		458	Y	Y	Y	Y	-2.245	0.122	2.28E-74	0.425	0.124	1.99E-02	0.617	0.124	4.18E-05	-0.177	0.126	3.21E-01	2564	720	535	823	472

NbUGT86A25	Group K	*not targeted*		488	Y	Y	Y	Y	3.129	0.284	2.86E-27	-0.425	0.256	4.83E-01	-0.391	0.256	4.33E-01	0.699	0.253	3.55E-02	32	231	313	236	516
NbUGT86A26	Group K	*not targeted*		499	Y	Y	Y	Y	-0.095	0.188	6.73E-01	-0.100	0.188	9.14E-01	-0.451	0.189	1.30E-01	-0.339	0.188	1.83E-01	603	525	564	410	443
NbUGT86A27	Group K	*not targeted*		498	Y	Y	Y	Y	-0.157	0.175	4.38E-01	-0.017	0.175	9.91E-01	-0.262	0.177	4.53E-01	-0.353	0.176	1.32E-01	355	314	318	264	247
NbUGT74B13	Group L	*not targeted*		464	Y	Y	Y	Y	5.702	0.375	6.49E-51	-2.539	0.329	6.68E-12	-1.838	0.327	1.75E-06	0.848	0.323	4.52E-02	10	128	825	214	1533
NbUGT74N5	Group L	*not targeted*		459	Y	Y	Y	Y	-0.437	0.320	2.24E-01	-0.104	0.320	9.54E-01	0.521	0.320	3.90E-01	1.545	0.320	2.85E-04	4116	2767	2987	4378	9270
NbUGT74P8	Group L	*not targeted*		446	Y	Y	Y	Y	-0.934	0.068	3.67E-42	0.095	0.069	6.10E-01	0.212	0.069	2.91E-02	0.199	0.068	2.73E-02	2985	1666	1561	1808	1793
NbUGT74P9	Group L	*not targeted*		450	Y	Y	Y	Y	-3.511	0.438	6.28E-15	0.381	0.449	8.21E-01	1.213	0.445	6.72E-02	-0.592	0.459	3.68E-01	402	36	27	70	16
NbUGT75A4	Group L	*not targeted*		474	Y	no	Y	Y	-1.449	0.242	6.89E-09	-0.124	0.242	9.19E-01	0.815	0.242	1.39E-02	1.080	0.242	7.75E-04	4119	1338	1462	2619	3165
NbUGT75A5	Group L	*not targeted*		482	Y	no	Y	Y	0.794	0.605	2.43E-01	-0.227	0.604	9.47E-01	0.660	0.603	6.27E-01	3.151	0.603	7.53E-05	57	100	125	230	1997
NbUGT75S2	Group L	*not targeted*		465	Y	no	Y	Y	5.089	0.456	7.47E-28	-0.036	0.421	9.92E-01	-0.173	0.421	8.95E-01	1.820	0.420	1.12E-03	7	473	486	426	1942
NbUGT84A75	Group L	*not targeted*		504	Y	no	Y	Y	0.874	0.670	2.46E-01	-0.542	0.669	8.34E-01	0.389	0.666	8.34E-01	3.112	0.664	4.22E-04	5	9	17	26	329
NbUGT84A76	Group L	*not targeted*		494	Y	no	Y	Y	3.938	0.491	5.64E-15	0.025	0.407	9.94E-01	0.089	0.407	9.51E-01	1.116	0.403	3.50E-02	2	81	79	85	184
NbUGT92G9	Group M	*not targeted*		496	Y	Y	Y	Y	-0.852	0.118	2.88E-12	0.121	0.121	7.65E-01	-0.081	0.122	8.04E-01	0.409	0.119	1.03E-02	705	423	389	368	519
NbUGT82E1	Group N	*not targeted*		441	Y	Y	Y	no	-1.868	0.197	1.85E-20	0.691	0.204	2.17E-02	0.923	0.203	2.34E-04	0.498	0.205	6.33E-02	282	123	75	145	108
NbUGT93S1	Group O	*not targeted*		476	Y	Y	Y	Y	-0.190	0.390	6.87E-01	0.475	0.391	NA	0.000	0.390	NA	0.000	0.390	1.00E+00	4	12	0	0	0
NbUGT93S2	Group O	*not targeted*		459	Y	Y	Y	Y	-1.744	0.319	1.35E-07	-0.337	0.328	7.56E-01	0.328	0.324	6.61E-01	1.081	0.320	1.15E-02	308	67	86	110	190
NbUGT93T1	Group O	*not targeted*		468	Y	Y	Y	Y	5.308	0.323	3.66E-59	0.498	0.297	4.77E-01	0.281	0.297	6.89E-01	1.315	0.297	8.65E-04	27	2065	1438	1763	3734
NbUGT93T2	Group O	*not targeted*		464	Y	Y	Y	Y	0.636	0.139	1.12E-05	-0.186	0.138	6.22E-01	-0.185	0.138	5.17E-01	-0.345	0.138	5.65E-02	1313	1799	2049	1801	1609
NbUGT93U1	Group O	*not targeted*		471	Y	Y	Y	Y	0.348	0.118	5.48E-03	-0.161	0.116	6.06E-01	-0.325	0.117	6.25E-02	-0.283	0.117	6.44E-02	479	547	612	488	502
NbUGT709B3	Group P	*not targeted*		471	no	Y	Y	Y	5.917	0.408	1.76E-46	0.051	0.238	9.74E-01	0.034	0.239	9.70E-01	-0.319	0.239	3.49E-01	2	296	286	293	227
NbUGT709J6	Group P	*not targeted*		475	Y	Y	Y	Y	-1.040	0.123	1.35E-16	0.029	0.123	9.70E-01	0.366	0.123	3.84E-02	0.458	0.123	5.27E-03	3540	1746	1712	2211	2359
NbUGT709L7	Group P	*not targeted*		531	no	Y	Y	Y	0.182	0.078	3.04E-02	-0.147	0.078	3.80E-01	-0.054	0.078	7.88E-01	0.069	0.077	5.60E-01	2174	2226	2466	2374	2587
NbUGT709Q1	Group P	*not targeted*		491	no	Y	Y	Y	0.861	0.097	5.27E-18	0.060	0.095	8.88E-01	0.181	0.095	2.79E-01	-0.154	0.095	2.43E-01	978	1858	1782	2021	1600
NbUGT709Q2	Group P	*not targeted*		381	no	Y	Y	Y	-0.491	0.272	1.01E-01	-0.040	0.273	9.84E-01	-0.544	0.274	2.47E-01	-0.801	0.274	2.68E-02	689	470	484	327	271
NbUGT709U2	Group P	*not targeted*		585	Y	Y	Y	Y	0.937	0.339	9.67E-03	0.383	0.334	7.09E-01	1.069	0.334	2.15E-02	1.435	0.333	1.20E-03	117	311	235	516	675
NbUGT95F1	undefined	*not targeted*		479	Y	Y	Y	no	-3.364	0.198	4.70E-63	0.711	0.205	1.77E-02	0.807	0.205	2.44E-03	-0.295	0.212	3.26E-01	1228	189	114	202	92

Supplementary Table 3 Reported activities of Family 1 UGTs groups targeted for Cas9-induced mutagenesis in *N. benthamiana*

T1 Line	Mutated NbUGT	Groups	Reported activities for this group
NbCas9-0780B-01	94E7	A	**Group A includes GTs that:**<br>- catalyze glycosidic branch elongation[2]<br>- catalyze the formation of di/tri-glycosides[3,4]<br>- are active on soyasaponins[5]
0720-06-01	73A24 73A25 73Q2 73A32	D	**Group D includes GTs that are active on:**<br>- geraniol[6]<br>- triterpenoids[2,6]<br>- flavonoids[7]<br>- benzoates[8]<br>- brassinosteroids[9]
0720-06-03	73A24 73A25 73Q2 73A32		
0724-37-06	72AY1 72AX1 72B34 72B58	E	**Group E includes GTs that are active on:**<br>- terpenoids[6] including geraniol[1]<br>- isoflavones[10]<br>- dihydrochalcones[11]<br>benzoates[12]
0724-22a-06	72AY1 72AX1 72B34 72B35		
NbCas9-0779A-01	71A57		
0361-34-05	85A73 85A74 86A104	G	**Group G includes GTs that are active on:**<br>- terpenoids[6] including geraniol[1]<br>- cytokinins[12]<br>- cyanogenic glucosides[13]<br>- abscisic acid[14]<br>-cyanohydrins[13]<br>-steviol[15]
0361-34-08	85A73 85A74 86A104		
NbCas9-0781B-03	72AY1 85A73	E, G	*see above*
NbCas9-0777B-01	74P7	L	**Group L includes GTs that are active on:**<br>- phenylpronpanoids[16]<br>- benzoates[7]<br>- auxin[17]<br>- hydroxycinnamates[2]
NbCas9-0778B-05	73E22 71A56 93T3	D, E, O	*Group D and E see above*<br>**Group O includes GTs that are active on:**<br>- cytokinins[18]

Supplementary Table 4 Coding sequences cloned into pEAQ-HT-DEST1

Enzyme		Organism	Key Reference	Accession #
CrDXS	1-deoxy-D-xylulose 5-phosphate synthase	*Catharanthus roseus* (Madagascar periwinkle)	this study	DQ848672
CrGPPS	geranyl diphosphate synthase large subunit	*Catharanthus roseus* (Madagascar periwinkle)	Rai et al (2013)[19]	JX417183
CrGES	geraniol synthase	*Catharanthus roseus* (Madagascar periwinkle)	Simkin et al (2013)[20]	JN882024
CrG8H	geraniol 8-oxidase	*Catharanthus roseus* (Madagascar periwinkle)	Collu et al (2001)[21]	AJ251269
CrGOR	8-hydroxygeraniol reductase	*Catharanthus roseus* (Madagascar periwinkle)	Miettinen et al (2014)[22] and Krithika et al (2015)[23]	KF302069
CrISY	iridoid synthase	*Catharanthus roseus* (Madagascar periwinkle)	Geu-Flores et al (2012)[24]	JX974564
GFP	green fluorescent protein	*Aequorea victoria* (crystal jelly)	Chalfie et al (1994)[25]	AF183395.1

Supplementary Table 5 Coding sequences cloned into pUAP1 (Addgene #63674) resulting in Level 0 standard parts. Asterisk indicates that the sequence encoding the native transit peptide was removed.

**Enzyme**		**Organism**	**Key reference(s)**	**Accession #**	**Level 0 plasmid name (AATG-GCTT)**	**Addgene #**
CrDXS*	1-deoxy-D-xylulose 5-phosphate synthase	*Catharanthus roseus* (Madagascar periwinkle)	*this study*	DQ848672	pEPQD0CM0065	177019
AstHMGR	truncated 3-hydroxy-3-methylglutaryl-coenzyme A reductase	*Avena strigosa* (oat)	Reed *et al.* (2017)[26]	KY284573	pL0-AstHMGR**	n/a - a gift from Anne Osbourn
PaGPPS*	geranyl pyrophosphate synthase; geranyl diphosphate synthase	*Picea abies* (Norway spruce)	Schmidt *et al.* (2010)[27]	GQ369788	pEPQD0CM0818	177020
CrGES*	geraniol synthase	*Catharanthus roseus* (Madagascar periwinkle)	Simkin *et al.* (2013)[20]	JN882024	pEPQD0CM0063	177021
CrG8H	geraniol 8-oxidase; geraniol-10-hydroxylase; CYP76B6	*Catharanthus roseus* (Madagascar periwinkle)	Collu *et al.* (2001)[21]	AJ251269	pEPQD0CM0058	177022
CrGOR	8-hydroxygeraniol oxidoreductase; 10-hydroxygeraniol oxidoreductase; alcohol dehydrogenase 10	*Catharanthus roseus* (Madagascar periwinkle)	Miettinen et al (2014)[22] and Krithika et al (2015)[23]	KF302069	pEPQD0CM0059	177023
CrISY	iridoid synthase	*Catharanthus roseus* (Madagascar periwinkle)	Geu-Flores *et al.* (2012)	JX974564	pEPQD0CM0060	177024
NmMLPL	major latex protein–like	*Nepeta mussinii (aka Nepeta racemosa)*	Lichman *et al.* (2020)[28] and Lichman *et al.* (2019)[29]	MT108267.1	pEPQD0CM0068	177025
CrIO	iridoid oxidase; CYP76A26	*Catharanthus roseus* (Madagascar periwinkle)	Miettinen et al (2014)[22]and Salim *et al.* (2014)[30]	KF302066	pEPQD0CM0061	177026
Cr7-DLGT	7-deoxyloganetic acid glucosyl transferase; UGT709C2	*Catharanthus roseus* (Madagascar periwinkle)	Miettinen et al (2014)[22]and Asada *et al.* (2013)[31]	KF302068	pEPQD0CM0062	177027
Cr7-DLH	7-deoxyloganic acid hydroxylase; CYP72A224	*Catharanthus roseus* (Madagascar periwinkle)	Miettinen et al (2014)[22] and Salim *et al* (2014)[30]	KF302067	pEPQD0CM0762	177028
CrLAMT	loganic acid O-methyltransferase	*Catharanthus roseus* (Madagascar periwinkle)	Murata *et al.* (2008)[32]	EU057974	pEPQD0CM0763	177029
CrSLS	secologanin synthase; CYP72C	*Catharanthus roseus* (Madagascar periwinkle)	Irmler *et al.* (2000)[33]	KF309242.1 or KF415117.1	pEPQD0CM0764	177030
CrTDC	tryptophan decarboxylase	*Catharanthus roseus* (Madagascar periwinkle)	de Luca *et al.* (1989)[34]	M25151	pEPQD0CM0765	177031
CrSTR	strictosidine synthase	*Catharanthus roseus* (Madagascar periwinkle)	Pasquali *et al.* (1992)[35]	X61932	pEPQD0CM0766	177032

Supplementary Table 6 Level 1 expression constructs assembled from Level 0 parts using the Plant MoClo Toolkit (Addgene Kit #1000000044)

	**(Promoter+5'UTR) *or* (Promoter)+(5'UTR+cTP)**		**CDS**	**3'UTR + Terminator**	**Level 1 Acceptor**
**overhangs created by BsaI:**	**GGAG-TACT**	**TACT-AATG**	**AATG-GCTT**	**GCTT-CGCT**	**CGCT-GGAG**
pEPQD1CB0104 (P6_35SshortTMV-P19-35Sterm)	pICH51277		pICH44022	pUAP41414	pICH47781
pEPQD1CB0817 (P1_35SshortTMV-AstHMGR-35Sterm)	pICH51277		pL0-AstHMGR	pUAP41414	pICH47732
pEPQD1CB0107 (P1 _35SshortTMV-cTP_CrDXS2-35Sterm)	pICH41388	pICH78133	pEPQD0CM0065	pUAP41414	pICH47732
pEPQD1CB0108 (P2_35SshortTMV-PaGPPS1-35Sterm)	pICH51277		pEPQD0CM0818	pUAP41414	pICH47742
pEPQD1CB0109 (P2_35SshortTMV-cTP_PaGPPS1-35Sterm)	pICH41388	pICH78133	pEPQD0CM0818	pUAP41414	pICH47742
pEPQD1CB0110 (P3_35SshortTMV-CrGES-35Sterm)	pICH51277		pEPQD0CM0063	pUAP41414	pICH47751
pEPQD1CB0112 (P3_35SshortTMV-cTP_CrGES-35Sterm)	pICH41388	pICH78133	pEPQD0CM0063	pUAP41414	pICH47751
pEPQD1CB0113 (P4_35SshortTMV-CrG8H-35Sterm)	pICH51277		pEPQD0CM0058	pUAP41414	pICH47761
pEPQD1CB0114 (P4_35SshortTMV-cTP_CrG8H-35Sterm)	pICH41388	pICH78133	pEPQD0CM0058	pUAP41414	pICH47761
pEPQD1CB0115 (P5_35SshortTMV-Cr8HGO/GOR-35Sterm)	pICH51277		pEPQD0CM0059	pUAP41414	pICH47772
pEPQD1CB0116 (P5_35SshortTMV-cTP_Cr8HGO/GOR-35Sterm)	pICH41388	pICH78133	pEPQD0CM0059	pUAP41414	pICH47772
pEPQD1CB0117 (P6_35SshortTMV-CrISY-35Sterm)	pICH51277		pEPQD0CM0060	pUAP41414	pICH47781
pEPQD1CB0118 (P6_35SshortTMV-cTP_CrISY-35Sterm)	pICH41388	pICH78133	pEPQD0CM0060	pUAP41414	pICH47781
pEPQD1CB0119 (P1 _35SshortTMV-NmMLP-35Sterm)	pICH51277		pEPQD0CM0068	pUAP41414	pICH47732
pEPQD1CB0120 (P1 _35SshortTMV-cTP_NmMLP-35Sterm)	pICH41388	pICH78133	pEPQD0CM0068	pUAP41414	pICH47732
pEPQD1CB0121 (P2_35SshortTMV-CrIO-35Sterm)	pICH51277		pEPQD0CM0061	pUAP41414	pICH47742
pEPQD1CB0122 (P2_35SshortTMV-cTP_CrIO-35Sterm)	pICH41388	pICH78133	pEPQD0CM0061	pUAP41414	pICH47742
pEPQD1CB0123 (P3_35SshortTMV-CrDLGT-35Sterm)	pICH51277		pEPQD0CM0062	pUAP41414	pICH47751
pEPQD1CB0124 (P3_35SshortTMV-cTP_CrDLGT-35Sterm)	pICH41388	pICH78133	pEPQD0CM0062	pUAP41414	pICH47751
pEPQD1CB0767 (P4_35SshortTMV-Cr7DLH-35Sterm)	pICH51277		pEPQD0CM0762	pUAP41414	pICH47761
pEPQD1CB0768 (P4_35SshortTMV-cTP_Cr7DLH-35Sterm)	pICH41388	pICH78133	pEPQD0CM0762	pUAP41414	pICH47761
pEPQD1CB0769 (P5_35SshortTMV- CrLAMT-35Sterm)	pICH51277		pEPQD0CM0763	pUAP41414	pICH47772
pEPQD1CB0770 (P5_35SshortTMV-cTP_ CrLAMT-35Sterm)	pICH41388	pICH78133	pEPQD0CM0763	pUAP41414	pICH47772
pEPQD1CB0771 (P6_35SshortTMV- CrSLS-35Sterm)	pICH51277		pEPQD0CM0764	pUAP41414	pICH47781
pEPQD1CB0772 (P6_35SshortTMV-cTP_ CrSLS-35Sterm)	pICH41388	pICH78133	pEPQD0CM0764	pUAP41414	pICH47781
pEPQD1CB0773 (P1_35SshortTMV- CrTDC-35Sterm)	pICH51277		pEPQD0CM0765	pUAP41414	pICH47732
pEPQD1CB0774 (P1_35SshortTMV-cTP_ CrTDC-35Sterm)	pICH41388	pICH78133	pEPQD0CM0765	pUAP41414	pICH47732
pEPQD1CB0775 (P2_35SshortTMV- CrSTR-35Sterm)	pICH51277		pEPQD0CM0766	pUAP41414	pICH47742
pEPQD1CB0776 (P2_35SshortTMV-cTP_ CrSTR-35Sterm)	pICH41388	pICH78133	pEPQD0CM0766	pUAP41414	pICH47742

Parts from Plant MoClo Parts Kit (Addgene Kit #1000000047)
pICH51277 (CaMV 35S short promoter + TMV omega 5'UTR) Addgene#50268
pICH41388 (CaMV 35S short promoter) Addgene#50253
pICH78133 (TMV omega 5'UTR+chloroplast transit peptide RbcS) Addgene#50292
pUAP41414 (CMV 3'UTR+terminator) Addgene#50337
pICH44022 (L0, P19 CDS) Addgene#50330
pICH47732 (L1 P1 acceptor forward) Addgene#48000
pICH47742 (L1 P2 acceptor forward) Addgene#48001
pICH47751 (L1 P3 acceptor forward) Addgene#48002
pICH47761 (L1 P4 acceptor forward) Addgene#48003
pICH47772 (L1 P5 acceptor forward) Addgene#48004
pICH47781 (L1 P6 acceptor forward) Addgene#48005

Abbreviations
CaMV = Cauliflower mosaic virus
TMV = Tobacco Mosaic Virus
P19 suppressor of gene silencing (Tomato Bushy Stunt Virus)
cTP = chloroplast transit peptide

Supplementary Table 7. Ratios of *A. tumefaciens* strains containing pEAQ plasmid vectors infiltrated into *N. benthamiana*

	**Low geraniol**	**High geraniol**	**Nepetalactol**	**Infiltration control**
**GFP**	6 units	1 unit	1 unit	1 unit
**CrDXS**		1 unit	1 unit	
**CrGGPPS.LSU**		1 unit	1 unit	
**CrGES**	1 unit	1 unit	1 unit	
**CrG8H**			1 unit	
**Cr8HGO/GOR**			1 unit	
**CrISY**			1 unit	

Supplementary Table 8. Family 1 UDP-glycosyltransferases (UGTs)

Enzyme	Group code	Organism	Genbank ID	Notes
AdGT4	Group G	*Actinidia deliciosa (kiwifruit)*	AIL51400	Activity on geraniol reported by Yauk *et al.* *Plant J.* 80:317–30 (2014)[36]
AtUGT79B1	Group A	*Arabidopsis thaliana*	AB018115, MJP23, 2800-4206	
AtUGT79B100	Group A	*Arabidopsis thaliana*	AC006193, 78745-80088	
AtUGT79B111	Group A	*Arabidopsis thaliana*	AC006193, 80861-82219	
AtUGT79B2	Group A	*Arabidopsis thaliana*	AL161571, 70597-71964	
AtUGT79B3	Group A	*Arabidopsis thaliana*	AL035602, T29A15, 13430-14791	
AtUGT79B4	Group A	*Arabidopsis thaliana*	AP000606, MTO24, 63467-64813	
AtUGT79B5	Group A	*Arabidopsis thaliana*	AC012561, F11F12, 44850-46196	
AtUGT79B6	Group A	*Arabidopsis thaliana*	AB007644, K19P17, 59557-60918	
AtUGT79B7	Group A	*Arabidopsis thaliana*	AC006567, T15G18, 32112-33440	
AtUGT79B8	Group A	*Arabidopsis thaliana*	AC004786, 55469-56797	
AtUGT79B9	Group A	*Arabidopsis thaliana*	AB007644, K19P17, 55445-56788	
AtUGT91A1	Group A	*Arabidopsis thaliana*	AC006340, 3826-5238	
AtUGT91B1	Group A	*Arabidopsis thaliana*	AB026639, 14713-16113	
AtUGT91C1	Group A	*Arabidopsis thaliana*	AB025613, 26973-28355	
AtUGT89A2	Group B	*Arabidopsis thaliana*	AL162751, 85361-86758	
AtUGT89B1	Group B	*Arabidopsis thaliana*	AC016662, F2P9, 86614-88035	
AtUGT89C1	Group B	*Arabidopsis thaliana*	AC024174, 19642-20949	
AtUGT90A1	Group C	*Arabidopsis thaliana*	AC005167, F12A24, 24146-26230	
AtUGT90A2	Group C	*Arabidopsis thaliana*	AC005489, F14N23, 95228-96717	
AtUGT90A4	Group C	*Arabidopsis thaliana*	AL391149, T9L3, 73006-74878	
AtUGT73B1	Group D	*Arabidopsis thaliana*	AL021961, F28A23, 51775-53366	
AtUGT73B2	Group D	*Arabidopsis thaliana*	AL021961, F28A23, 48984-50524	
AtUGT73B3	Group D	*Arabidopsis thaliana*	AL021961, F28A23, 176825-178270	
AtUGT73B4	Group D	*Arabidopsis thaliana*	AC006248, F26H6, 11828-13404	
AtUGT73B5	Group D	*Arabidopsis thaliana*	AC006248, F26H6, 8823-10458	
AtUGT73C1	Group D	*Arabidopsis thaliana*	AC006282, F13K3, 61903-63378	Low activity on geraniol (10-40%), reported by Caputi *et al.* (2008)[6]
AtUGT73C2	Group D	*Arabidopsis thaliana*	AC006282, F13K3, 64414-65904	
AtUGT73C3	Group D	*Arabidopsis thaliana*	AC006282, F13K3, 68990-70480	Very low activity on geraniol (1-10%), reported by Caputi *et al.* (2008)[6]
AtUGT73C4	Group D	*Arabidopsis thaliana*	AC006282, F13K3, 66599-68089	
AtUGT73C5	Group D	*Arabidopsis thaliana*	AC006282, F13K3, 74865-76352	High activity on geraniol (>40%) reported by Caputi *et al.* (2008)[6]
AtUGT73C6	Group D	*Arabidopsis thaliana*	AC006282, F13K3, 71711-73198	Low activity on geraniol (10-40%), reported by Caputi *et al.* (2008)[6]
AtUGT73C7	Group D	*Arabidopsis thaliana*	AL132958, T4D2, 26553-28025	
AtUGT73D1	Group D	*Arabidopsis thaliana*	AL132958, T4D2, 21804-23327	
AtUGT71B1	Group E	*Arabidopsis thaliana*	AB025634, MSD21, 21447-22865	

Enzyme	Group code	Organism	Genbank ID	Notes
AtUGT71B2	Group E	*Arabidopsis thaliana*	AB025634, MSD21, 23981-25438	
AtUGT71B5	Group E	*Arabidopsis thaliana*	AL161541, 37302-38738	
AtUGT71B6	Group E	*Arabidopsis thaliana*	AB025634, MSD21, 31933-33372	
AtUGT71B7	Group E	*Arabidopsis thaliana*	AB025634, MSD21, 33809-35296	
AtUGT71B8	Group E	*Arabidopsis thaliana*	AB025634, MSD21, 37125-38567	
AtUGT71C1	Group E	*Arabidopsis thaliana*	AC005496, T27A16, 51968-53413	
AtUGT71C2	Group E	*Arabidopsis thaliana*	AC005496, T27A16, 48813-50237	Very low activity on geraniol (1-10%), reported by Caputi *et al.* (2008)[6]
AtUGT71C3	Group E	*Arabidopsis thaliana*	AC067971, F10K1.3, 12382-13812	
AtUGT71C4	Group E	*Arabidopsis thaliana*	AC067971, F10K1.4, 14158-15597	
AtUGT71C5	Group E	*Arabidopsis thaliana*	AC067971, F10K1, 16229-17671	
AtUGT71D1	Group E	*Arabidopsis thaliana*	AC005496, T27A16, 45718-47121	
AtUGT71D2	Group E	*Arabidopsis thaliana*	AC005496, T27A16, 40783-42186	
AtUGT72B1	Group E	*Arabidopsis thaliana*	AL161491, 75572-77014	
AtUGT72B2	Group E	*Arabidopsis thaliana*	AC023628, F6F3.19, 89227-90669	
AtUGT72B3	Group E	*Arabidopsis thaliana*	AC023628, F6F3.22, 95474-96919	
AtUGT72C1	Group E	*Arabidopsis thaliana*	AL161590, 17779-19152	
AtUGT72D1	Group E	*Arabidopsis thaliana*	AC006135, F24H14, 59343-60755	
AtUGT72E1	Group E	*Arabidopsis thaliana*	AL132979, T3A5, 67179-68615	
AtUGT72E2	Group E	*Arabidopsis thaliana*	AB018119, MSN2, 30560-32005	
AtUGT72E3	Group E	*Arabidopsis thaliana*	AF077407, F9D12, 79017-80462	
AtUGT88A1	Group E	*Arabidopsis thaliana*	AP000373, 56926-58404	Low activity on geraniol (10-40%), reported by Caputi *et al.* (2008)[6]
AtUGT78D1	Group F	*Arabidopsis thaliana*	AC009917, F26G16, 65767-67224	
AtUGT78D2	Group F	*Arabidopsis thaliana*	AL391141, F2K13, 64922-66486	
AtUGT78D3	Group F	*Arabidopsis thaliana*	AL391141, F2K13, 60292-61817	
AtUGT85A1	Group G	*Arabidopsis thaliana*	AC006551, F12K8, 101428-104184	High activity on geraniol (>40%) reported by Caputi *et al.* (2008)[6]
AtUGT85A2	Group G	*Arabidopsis thaliana*	AC068562, 3508-5967	High activity on geraniol (>40%) reported by Caputi *et al.* (2008)[6]
AtUGT85A3	Group G	*Arabidopsis thaliana*	AC006551, F12K8, 105703-107198; AC068562, T16E15,	
AtUGT85A4	Group G	*Arabidopsis thaliana*	AC013430, 74211-75743	High activity on geraniol (>40%) reported by Caputi *et al.* (2008)[6]
AtUGT85A5	Group G	*Arabidopsis thaliana*	AC068562, T16E15.2, 1156-2919	Very low activity on geraniol (1-10%), reported by Caputi *et al.* (2008)[6]
AtUGT85A7	Group G	*Arabidopsis thaliana*	AC068562, T16E15.5, 8945-10571	High activity on geraniol (>40%) reported by Caputi *et al.* (2008)[6]
AtUGT76B1	Group H	*Arabidopsis thaliana*	AC073395, F11B9, 93894-95312	
AtUGT76C1	Group H	*Arabidopsis thaliana*	AB017060, K18J17	

**Enzyme**	**Group code**	**Organism**	**Genbank ID**	**Notes**
AtUGT76C2	Group H	*Arabidopsis thaliana*	AB005237, MJJ3, 86268-87835; AB017060, K18J17, 1-2	
AtUGT76C3	Group H	*Arabidopsis thaliana*	AB017060, K18J17, 7401-9269	
AtUGT76C4	Group H	*Arabidopsis thaliana*	AB017060, K18J17, 2536-4403	
AtUGT76C5	Group H	*Arabidopsis thaliana*	AB017060, K18J17, 5455-6900	
AtUGT76D1	Group H	*Arabidopsis thaliana*	AC002505, T9J22, 49628-51237	Low activity on geraniol (10-40%), reported by Caputi *et al.* (2008)[6]
AtUGT76E1	Group H	*Arabidopsis thaliana*	AB025604, F2O15, 69708-71158	
AtUGT76E111	Group H	*Arabidopsis thaliana*	AL133314, F12A12, 91897-93329	Very low activity on geraniol (1-10%), reported by Caputi *et al.* (2008)[6]
AtUGT76E122	Group H	*Arabidopsis thaliana*	AL133314, F12A12, 88508-89949	High activity on geraniol (>40%) reported by Caputi *et al.* (2008)[6]
AtUGT76E2	Group H	*Arabidopsis thaliana*	AB025604, F2O15, 72621-74054	High activity on geraniol (>40%) reported by Caputi *et al.* (2008)[6]
AtUGT76E3	Group H	*Arabidopsis thaliana*	AL096859, T6H20, 92466-93881	
AtUGT76E4	Group H	*Arabidopsis thaliana*	AL133314, F12A12, 96862-98299	
AtUGT76E5	Group H	*Arabidopsis thaliana*	AL096859, T6H20, 81966-83384	
AtUGT76E6	Group H	*Arabidopsis thaliana*	AL133314, F12A12, 94420-95845	
AtUGT76E7	Group H	*Arabidopsis thaliana*	AB028606, F16F17, 34184-35615	
AtUGT76E9	Group H	*Arabidopsis thaliana*	AB028606, F16F17, 7449-9225	
AtUGT76F1	Group H	*Arabidopsis thaliana*	AL161667, F1I16, 61935-63899	
AtUGT76F2	Group H	*Arabidopsis thaliana*	AL161667, F1I16, 59290-61366	
AtUGT83A1	Group I	*Arabidopsis thaliana*	AC011664, F1C9, 38853-40497	
AtUGT87A1	Group J	*Arabidopsis thaliana*	AC004165, T27E13, 50431-51895	
AtUGT87A2	Group J	*Arabidopsis thaliana*	AC004165, 47973-49464	
AtUGT86A1	Group K	*Arabidopsis thaliana*	AC006922, T1J8, 88046-89708	
AtUGT86A2	Group K	*Arabidopsis thaliana*	AC005851, 28144-30597	
**AtUGT74B1**	**Group L**	*Arabidopsis thaliana*	AC002396, F3I6, 4859-6322	
**AtUGT74C1**	**Group L**	*Arabidopsis thaliana*	AC006533, T9H9, 34299-36197	
**AtUGT74D1**	**Group L**	*Arabidopsis thaliana*	AC006533, T9H9, 13342-15897	
**AtUGT74E1**	**Group L**	*Arabidopsis thaliana*	AC007153, F3F20, 82737-84239	
**AtUGT74E2**	**Group L**	*Arabidopsis thaliana*	AC007153, F3F20, 84720-86163	
**AtUGT74F1**	**Group L**	*Arabidopsis thaliana*	AC002333, F18O19, 82534-84019	
**AtUGT74F2**	**Group L**	*Arabidopsis thaliana*	AC002333, F18O19, 77132-78568	
**AtUGT75B1**	**Group L**	*Arabidopsis thaliana*	AC007153, F3F20, 27198-28607	
**AtUGT75B2**	**Group L**	*Arabidopsis thaliana*	AC007153, F3F20, 18020-19387	
**AtUGT75C1**	**Group L**	*Arabidopsis thaliana*	AL161538, 19874-21244	
**AtUGT75D1**	**Group L**	*Arabidopsis thaliana*	AL161542, 3576-5000	
**AtUGT84A1**	**Group L**	*Arabidopsis thaliana*	AL161541, 167121-168572	

Enzyme	Group code	Organism	Genbank ID	Notes
AtUGT84A2	Group L	*Arabidopsis thaliana*	AB019232, MIL23, 33583-35070	
AtUGT84A3	Group L	*Arabidopsis thaliana*	AL161541, 170982-172421	
AtUGT84A4	Group L	*Arabidopsis thaliana*	AL161541, 175213-176640	
AtUGT84B1	Group L	*Arabidopsis thaliana*	AC002391, F21P24, 58701-60071	
AtUGT84B2	Group L	*Arabidopsis thaliana*	AC002391, F21P24, 56464-57816	
AtUGT92A1	Group M	*Arabidopsis thaliana*	AL353013, T24H18, 21475-22941	
AtUGT82A1	Group N	*Arabidopsis thaliana*	AP002046, MMP21, 6263-8510	
CsUGT85K11	Group G	*Camellia sinensis (tea)*	BAO51834	Activity on geraniol reported by Ohgami *et al.* (2015)[37]
CrUGT709C2 (7-DLGT)	Group P	*Catharanthus roseus (Madagascar periwinkle)*	BAO01109.1	Iridoid activity reported by Asada *et al.* (2013)[31]
GjUGT85A24	Group G	*Gardenia jasminoides (Cape jasmine)*	BAK55737.1	Iridoid activity reported by Nagatoshi *et al.* (2011)[38]
NbUGT79A16	Group A	*Nicotiana benthamiana*	MT945391	
NbUGT91A20	Group A	*Nicotiana benthamiana*	MT945398	
NbUGT91R10	Group A	*Nicotiana benthamiana*	MT945403	
NbUGT91S4	Group A	*Nicotiana benthamiana*	MT945363	
NbUGT94AQ1	Group A	*Nicotiana benthamiana*	MT945339	
NbUGT94AR1	Group A	*Nicotiana benthamiana*	MT945389	
NbUGT94E7	Group A	*Nicotiana benthamiana*	MT945325	
NbUGT94U2	Group A	*Nicotiana benthamiana*	MT945381	
NbUGT89D20	Group B	*Nicotiana benthamiana*	MT945364	
NbUGT89V1	Group B	*Nicotiana benthamiana*	MT945356	
NbUGT90A22	Group C	*Nicotiana benthamiana*	MT945366	
NbUGT90K1	Group C	*Nicotiana benthamiana*	MT945337	
NbUGT73A24	Group D	*Nicotiana benthamiana*	MT945326	High activity on geraniol (81%) reported by Sun *et al.* (2019)[1]
NbUGT73A25	Group D	*Nicotiana benthamiana*	MT945327	High activity on geraniol (47%) reported by Sun *et al.* (2019)[1]
NbUGT73A32	Group D	*Nicotiana benthamiana*	MT945371	
NbUGT73AB10	Group D	*Nicotiana benthamiana*	MT945365	
NbUGT73BY1	Group D	*Nicotiana benthamiana*	MT945354	
NbUGT73BZ1	Group D	*Nicotiana benthamiana*	MT945359	
NbUGT73E20	Group D	*Nicotiana benthamiana*	MT945362	
NbUGT73E21	Group D	*Nicotiana benthamiana*	MT945336	
NbUGT73E22	Group D	*Nicotiana benthamiana*	MT945368	
NbUGT73E23	Group D	*Nicotiana benthamiana*	MT945372	
NbUGT73Q2	Group D	*Nicotiana benthamiana*	MT945333	
NbUGT71A56	Group E	*Nicotiana benthamiana*	MT945345	
NbUGT71A57	Group E	*Nicotiana benthamiana*	MT945369	
NbUGT71AJ1	Group E	*Nicotiana benthamiana*	MT945347	Very low activity on geraniol (1%) reported by Sun *et al.* (2019)[1]
NbUGT71AT2	Group E	*Nicotiana benthamiana*	MT945342	

Enzyme	Group code	Organism	Genbank ID	Notes
NbUGT71AT3	Group E	*Nicotiana benthamiana*	MT945324	
NbUGT71AT4	Group E	*Nicotiana benthamiana*	MT945343	
NbUGT71AT5	Group E	*Nicotiana benthamiana*	MT945386	
NbUGT71AU1	Group E	*Nicotiana benthamiana*	MT945367	
NbUGT71AV1	Group E	*Nicotiana benthamiana*	MT945376	
NbUGT71X3	Group E	*Nicotiana benthamiana*	MT945350	
NbUGT71X4	Group E	*Nicotiana benthamiana*	MT945358	
NbUGT72AX1	Group E	*Nicotiana benthamiana*	MT945344	Very low activity on geraniol (2%) reported by Sun *et al.* (2019)[1]
NbUGT72AY1	Group E	*Nicotiana benthamiana*	MT945401	Low activity on geraniol (23%) reported by Sun *et al.* (2019)[1]
NbUGT72B34*G322V	Group E	*Nicotiana benthamiana*	MT945340	Very low activity on geraniol (11%) reported by Sun *et al.* (2019)[1]
NbUGT72B35	Group E	*Nicotiana benthamiana*	MT945341	Very low activity on geraniol (1%) reported by Sun *et al.* (2019)[1]
NbUGT72B58	Group E	*Nicotiana benthamiana*	MT945379	
NbUGT78C2	Group F	*Nicotiana benthamiana*	MT945402	
NbUGT85A104	Group G	*Nicotiana benthamiana*	MT945331	
NbUGT85A73	Group G	*Nicotiana benthamiana*	MT945328	High activity on geraniol (100%) reported by Sun *et al.* (2019)[1]
NbUGT85A74	Group G	*Nicotiana benthamiana*	MT945370	Reported as inactive by Sun *et al.* (2019)[1]
NbUGT76A4	Group H	*Nicotiana benthamiana*	MT945330	
NbUGT76A5	Group H	*Nicotiana benthamiana*	MT945393	
NbUGT87AB1	Group J	*Nicotiana benthamiana*	MT945378	
NbUGT86A25	Group K	*Nicotiana benthamiana*	MT945374	
NbUGT86A26	Group K	*Nicotiana benthamiana*	MT945380	
NbUGT86A27	Group K	*Nicotiana benthamiana*	MT945388	
NbUGT74B13	Group L	*Nicotiana benthamiana*	MT945360	
NbUGT74N4	Group L	*Nicotiana benthamiana*	MT945323	
NbUGT74N5	Group L	*Nicotiana benthamiana*	MT945334	
NbUGT74N6	Group L	*Nicotiana benthamiana*	MT945361	
NbUGT74P7	Group L	*Nicotiana benthamiana*	MT945332	
NbUGT74P8	Group L	*Nicotiana benthamiana*	MT945355	
NbUGT74P9	Group L	*Nicotiana benthamiana*	MT945405	
NbUGT74T6	Group L	*Nicotiana benthamiana*	MT945322	
NbUGT75A4	Group L	*Nicotiana benthamiana*	MT945348	
NbUGT75A5	Group L	*Nicotiana benthamiana*	MT945351	
NbUGT75S2	Group L	*Nicotiana benthamiana*	MT945353	
NbUGT84A75	Group L	*Nicotiana benthamiana*	MT945384	
NbUGT84A76	Group L	*Nicotiana benthamiana*	MT945395	
NbUGT92G9	Group M	*Nicotiana benthamiana*	MT945373	
NbUGT82E1	Group N	*Nicotiana benthamiana*	MT945399	
NbUGT93S1	Group O	*Nicotiana benthamiana*	MT945346	
NbUGT93S2	Group O	*Nicotiana benthamiana*	MT945394	

Enzyme	Group code	Organism	Genbank ID	Notes
**NbUGT93T1**	**Group O**	*Nicotiana benthamiana*	MT945352	
**NbUGT93T2**	**Group O**	*Nicotiana benthamiana*	MT945357	
**NbUGT93T3**	**Group O**	*Nicotiana benthamiana*	MT945382	
**NbUGT93U1**	**Group O**	*Nicotiana benthamiana*	MT945375	
**NbUGT709B3**	**Group P**	*Nicotiana benthamiana*	MT945392	
**NbUGT709J6**	**Group P**	*Nicotiana benthamiana*	MT945329	
**NbUGT709L7**	**Group P**	*Nicotiana benthamiana*	MT945349	
**NbUGT709Q1**	**Group P**	*Nicotiana benthamiana*	MT945377	Reported as inactive by Sun *et al.* (2019)[1]
**NbUGT709Q2**	**Group P**	*Nicotiana benthamiana*	MT945387	
**NbUGT709U2**	**Group P**	*Nicotiana benthamiana*	MT945407	
NbUGT95F1	undefined	*Nicotiana benthamiana*	MT945404	
SbUGT95B1	undefined	*Sorghum bicolor*	AAF17077.1	Activity on geraniol reported by Jones *et al.* (1999)[39]
VvGT7	Group E	*Vitus vinifera (grape)*	XP_002276546	Activity on geraniol reported by Bönisch *et al.* (2014)[40]
**VvGT14**	**Group G**	*Vitus vinifera (grape)*	ENA: CCB45585.1	Activity on geraniol reported by Bönisch *et al.* (2014)[40]
**VvGT16**	**Group G**	*Vitus vinifera (grape)*	ENA: CCB58004.1	Activity on geraniol reported by Bönisch *et al.* (2014)[40]
VvGT15	Group H	*Vitus vinifera (grape)*	ENA: CCB43518.1	Activity on geraniol reported by Bönisch *et al.* (2014)[40]

Supplementary Table 9. Primers used for amplification of sgRNA scaffolds from pEPOR1CB0022 Addgene#117537 which contains the sgRNA stem extension scaffold sequence first reported by Chen *et al. Cell* 155(7):1479–91 (2013)[41]. Details of the U6-promoter and L1 acceptor that the resulting PCR amplicon was assembled with are also provided.

Construct Name	sgRNA #	target UGT	L1 acceptor	U6 Promoter	F primer	R primer
pEPQDPKN0361	2	NbUGT85104	Position 2 pICH47742 Addgene#48001	pICSL90002 (U6-26, A thaliana) Addgene#68261	tgtGGTCTCtattgTGATATGAAAGCCTCGTATAgtttaagagctatgctggaaac	tGGTCTCtagcgaaaaaaagcaccgact (for all amplifications)
	3	NbUGT85A73	Position 3 pICH47751 Addgene#48002	pEPQD0CM0032 (pUAP-NbU6-1) Addgene#185623	tgtGGTCTCtttcgACCCGTCTCGTGCATCGTGTgtttaagagctatgctggaaac	
	4	NbUGT85A74	Position 4 pICH47761 Addgene#48003	pEPQD0CM0033 (pUAP-NbU6-2) Addgene#185624	tgtGGTCTCtctcgAGAGGACCTTATTCTCTTAAgtttaagagctatgctggaaac	
	6	NbUGT85A73	Position 6 pICH47781 Addgene#48005	pICSL90002 (U6-26, A thaliana) Addgene#68261	tgtGGTCTCtattgCGTGGCCCTGATTCTCTCAAgtttaagagctatgctggaaac	
	7	NbUGT85104	Position 7 pICH47791 Addgene#48006	pEPQD0CM0032 (pUAP-NbU6-1) Addgene#185623	tgtGGTCTCtttcgTGTGTCGCGTCATCGTTAGAgtttaagagctatgctggaaac	
	1	NbUGT85A74	Position 1 pICH47732 Addgene#48000	pICSL90002 (U6-26, A thaliana) Addgene#68261	tgtGGTCTCtattGAGAAATGGGTTCTGTTGAAgtttaagagctatgctggaaac	
pEPQDPKN0720	2	NbUGT73Q2	Position 2 pICH47742 Addgene#48001	pICSL90002 (U6-26, A thaliana) Addgene#68261	tgtGGTCTCtattGTTGACGTTGCAGCCAAGCTgtttaagagctatgctggaaac	
	3	NbUGT73A24 and NbUGT73A25 and (*NbUGT73A32 - 2 bp mismatch)*	Position 3 pICH47751 Addgene#48002	pEPQD0CM0032 (pUAP-NbU6-1) Addgene#185623	tgtGGTCTCtttcGTGCCATGGAAAACTATTCTgtttaagagctatgctggaaac	
	4	NbUGT73A24 and NbUGT73A32 *(NbUGT73A25 - 2 bp mismatch)*	Position 4 pICH47761 Addgene#48003	pEPQD0CM0033 (pUAP-NbU6-2) Addgene#185624	tgtGGTCTCtctcGCTGCAGAATCAGTAGTCCAgtttaagagctatgctggaaac	
	6	NbUGT73A24 and NbUGT73A25 and *(NbUGT73A32 - 1 bp mismatch)*	Position 6 pICH47781 Addgene#48005	pICSL90002 (U6-26, A thaliana) Addgene#68261	tgtGGTCTCtattgTTAATTTCGTGAGGCAAATTgtttaagagctatgctggaaac	
	7	NbUGT73Q2	Position 7 pICH47791 Addgene#48006	pEPQD0CM0032 (pUAP-NbU6-1) Addgene#185623	tgtGGTCTCtttcGAGAATTTAGGTGCGTTGAGgtttaagagctatgctggaaac	
	1	NbUGT73A24 and NbUGT73A25	Position 1 pICH47732 Addgene#48000	pICSL90002 (U6-26, A thaliana) Addgene#68261	tgtGGTCTCtattgAGCTTCGCCATGTCTAGTGTgtttaagagctatgctggaaac	
pEPQDPKN0724	2	NbUGT72B34 and NbUGT72B35 and *(NbUGT72B58 - 1 bp mismatch)*	Position 2 pICH47742 Addgene#48001	pICSL90002 (U6-26, A thaliana) Addgene#68261	tgtGGTCTCtattgTGAGCTTGTGGGGCCCAATTgtttaagagctatgctggaaac	
	3	NbUGT72AY1	Position 3 pICH47751 Addgene#48002	pEPQD0CM0032 (pUAP-NbU6-1) Addgene#185623	tgtGGTCTCtttcGAAAACCACTCTCACCAATGgtttaagagctatgctggaaac	
	4	NbUGT72AX1	Position 4 pICH47761 Addgene#48003	pEPQD0CM0033 (pUAP-NbU6-2) Addgene#185624	tgtGGTCTCtctcGGATGATGTGTCCCATGCCAgtttaagagctatgctggaaac	
	6	NbUGT72AY1	Position 6 pICH47781 Addgene#48005	pICSL90002 (U6-26, A thaliana) Addgene#68261	tgtGGTCTCtattGGTTGCCTAAGACTAGAACTgtttaagagctatgctggaaac	
	7	NbUGT72B34 and NbUGT72B35	Position 7 pICH47791 Addgene#48006	pEPQD0CM0032 (pUAP-NbU6-1) Addgene#185623	tgtGGTCTCtttcGTTTCAATTTTAACATCAAGgtttaagagctatgctggaaac	
	1	NbUGT72B58	Position 1 pICH47732 Addgene#48000	pICSL90002 (U6-26, A thaliana) Addgene#68261	tgtGGTCTCtattgTTAGAGATGGGACCGTTAGTgtttaagagctatgctggaaac	

Supplementary Table 10. Primers used for construction of mobile single guide RNA plasmid vectors. pEPQDKN0761 (Addgene #185630) containing an sgRNA fused to truncated flowering locus T was used as a PCR template.

Construct	Target UGT	Position in final construct	F Primer	R Primer
pEPQDKN0777	NbUGT71AT3	1	aCGTCTCgcaggcacctgcaacgAAACTTTTGAACACCATAGTCTAAgttttagagctag	tCGTCTCccgagtcacctgctagtGCACgctcaacacgtacccggccgcgattggccataagtaacctttagagt
	NbUGT74P7	2	aCGTCTCgcaggcacctgcaacgGTGCGATTTATGTGGCCTTGTGCGgttttagagctag	tCGTCTCccgagtcacctgctagtCCTGgctcaacacgtacccggccgcgattggccataagtaacctttagagt
	NbUGT90K1	3	aCGTCTCgcaggcacctgcaacgCAGGGGTGGCGCAAAAGGGCCGCGgttttagagctag	tCGTCTCccgagcacctgctagtCACTttggccataagtaacctttt
pEPQDKN0778	NbUGT71A56	1	aCGTCTCgcaggcacctgcaacgAAACTCACCTTGTACCCACTGTGGgttttagagctag	tCGTCTCccgagtcacctgctagtGCACgctcaacacgtacccggccgcgattggccataagtaacctttagagt
	NbUGT73E22	2	aCGTCTCgcaggcacctgcaacgGTGCATCGCGCAGTGTCAATCATAgttttagagctag	tCGTCTCccgagtcacctgctagtCCTGgctcaacacgtacccggccgcgattggccataagtaacctttagagt
	NbUGT93T3	3	aCGTCTCgcaggcacctgcaacgCAGGGTAAACAGGAAGACCATAAGgttttagagctag	tCGTCTCccgagcacctgctagtCACTttggccataagtaacctttt
pEPQDKN0779	NbUGT73BY1	1	aCGTCTCgcaggcacctgcaacgAAACCCACACCTCTCAATGCCCAAgttttagagctag	tCGTCTCccgagtcacctgctagtGCACgctcaacacgtacccggccgcgattggccataagtaacctttagagt
	NbUGT74N6	2	aCGTCTCgcaggcacctgcaacgGTGCCTACCAAGCGGAAAGCTTCGgttttagagctag	tCGTCTCccgagtcacctgctagtCCTGgctcaacacgtacccggccgcgattggccataagtaacctttagagt
	NbUGT71A57	3	aCGTCTCgcaggcacctgcaacgCAGGCTTATGACCGGCAATAAACGgttttagagctag	tCGTCTCccgagcacctgctagtCACTttggccataagtaacctttt
pEPQDKN0780	NbUGT74T6	1	aCGTCTCgcaggcacctgcaacgAAACGTATCGATACCATTTCCGATgttttagagctag	tCGTCTCccgagtcacctgctagtGCACgctcaacacgtacccggccgcgattggccataagtaacctttagagt
	NbUGT74N4	2	aCGTCTCgcaggcacctgcaacgGTGCCATAAACTATGCAATTCACAgttttagagctag	tCGTCTCccgagtcacctgctagtCCTGgctcaacacgtacccggccgcgattggccataagtaacctttagagt
	NbUGT94E7	3	aCGTCTCgcaggcacctgcaacgCAGGCTAAGGGCGTGATTGAGATGgttttagagctag	tCGTCTCccgagcacctgctagtCACTttggccataagtaacctttt
pEPQDKN0781	NbUGT85A73	1	aCGTCTCgcaggcacctgcaacgAAACCGTGGCCCTGATTCTCTCAAgttttagagctag	tCGTCTCccgagtcacctgctagtGCACgctcaacacgtacccggccgcgattggccataagtaacctttagagt
	NbUGT73A24 and NbUGT73A25	2	aCGTCTCgcaggcacctgcaacgGTGCAGCTTCGCCATGTCTAGTGTgttttagagctag	tCGTCTCccgagtcacctgctagtCCTGgctcaacacgtacccggccgcgattggccataagtaacctttagagt
	NbUGT72AY1	3	aCGTCTCgcaggcacctgcaacgCAGGGGTTGCCTAAGACTAGAACTgttttagagctag	tCGTCTCccgagcacctgctagtCACTttggccataagtaacctttt

Supplementary Table 11. Primers used for genotyping plants with Cas9-induced mutations. (A) Primers used for PCR amplification and sequencing of UGT target genes (B) Primers used for ddPCR amplification to determine T-DNA copy number. Asterisk indicates phosphorothioate bond.

A

UGT	Group	Sequencing primer (forward)	Sequencing primer (reverse)	Sequencing primer (alternate rev.)
NbUGT85A73	G	caactcaaatcaactgtgaattttcc	gggagcagcctatatatgtcgg	
NbUGT85A74		aattgaaaatattgtctatcgaaagagg	aatacccatttgttatatcacttgtatctg	
NbUGT85A104		ccaaaactgcctttgaacaatc	cgtacctttcactggacaataacc	
NbUGT73A24	D	tctattttctgccactaaagcagg	tccactgaatcgaaccaaca*c	ctcttccccagatcgctc*g
NbUGT73A25		acacagctttacttttctttctgctac	gttctcaaaacctcagtcactaactt*c	tctcttccccagattgctc*a
NbUGT73Q2		ctactgcagttttttcttctcatcag	gaactcctatctttaaaatctctacgagc	
NbUGT73A32		aataagaactgaaactacatctagcagtagc	atataagtccatatatcttacatgccattg	tgactgactcttcatccgatag*g
NbUGT72B34*G322V	E	cctccctcctaatcttattgctat*c	cagaagtagacacatacagtttggc	gaatctgaatcgggtcaggtaag
NbUGT72B35		cgcaaataaatcagcgtaattttactag	gcacaattactaccagcttagaacaac	tgaaccgggtcggttacc
NbUGT72AX1		taattaaacaaatcgcacaacagtatc	atccaggagtttgaataggttc*g	
NbUGT72B58 (first exon)		aagaaaagtcttgtttagcaagcag	ggtccagaagttcctttgctt*c	
NbUGT72B58 (second exon)		ccctctcaaatacacttggatcag	acaagaacaaaacccatgtgctag	
NbUGT72AY1		gtagtttgtgaaattgtttatggcttc	cgaattgtaacattacctcgtcattc	
NbUGT71AT3	E	gatgaatgaactaattttcattcctttag	ggaacaatctcagaatccattaaagc	
NbUGT74P7	L	ttagtacataaaagatgacctatggtgc	aaggcaacgaaacaggaacag	
NbUGT90K1	C	actgctatatacataagaccgcgttc	gaaaacgtaaaaggcacgtctg	
NbUGT71A56	E	caaaacatccactgcaacatagtc	cataacttgggattccaaactcg	
NbUGT73E22	D	gtatcaaacaatggctgttgtca*g	gaatgttaaatttttgggcaaca*c	
NbUGT93T3	O	cttctatgggtagctgtgagaatttac	ttaagcaattcttcctcaagttg*g	
NbUGT73BY1	D	caaatccctttaataaaaatccatgc	tgaactcaatcttgtgtggca*c	
NbUGT74N6	L	ctcctcattcataattggccatag	aattgattcaccaacatttcaagt*g	
NbUGT71A57	E	gcaaagaagaaaatggaagatatcaag	acgacttcctatcaagaaatgtgtg	
NbUGT74T6	L	attttagctcttccttatccaagcc	tttaacaccaactcaaaatacgctg	
NbUGT74N4	L	gcttgatcttgccatatccagt*g	ggtacatctgaactctcaattgtacttg	
NbUGT94E7	A	catggatacacaagtaatagaatgtggt	tctttttctttagctgattttgcc	

B

gene	ddPCR primer (forward)	ddPCR primer (reverse)
nptII	cctgccgagaaagtatccat	tcttcgtccagatcatcctg
N. benthamiana Rdr1 (reference gene)	gttacgccatccatgtgttg	cagagttcaatttgccagca

Supplementary References

1. Sun, G. *et al.* Glucosylation of the phytoalexin N-feruloyl tyramine modulates the levels of pathogen-responsive metabolites in Nicotiana benthamiana. *Plant J.* **100**, 20–37 (2019).
2. Louveau, T. & Osbourn, A. The sweet side of plant-specialized metabolism. *Cold Spring Harb. Perspect. Biol.* **11**, a034744 (2019).
3. Thodberg, S. *et al.* Elucidation of the amygdalin pathway reveals the metabolic basis of bitter and sweet almonds (Prunus dulcis). *Plant Physiol.* **178**, 1096–1111 (2018).
4. Noguchi, A. *et al.* Local differentiation of sugar donor specificity of flavonoid glycosyltransferase in Lamiales. *Plant Cell* **21**, 1556–1572 (2009).
5. Shibuya, M., Nishimura, K., Yasuyama, N. & Ebizuka, Y. Identification and characterization of glycosyltransferases involved in the biosynthesis of soyasaponin I in Glycine max. *FEBS Lett.* **584**, 2258–2264 (2010).
6. Caputi, L., Lim, E.-K. & Bowles, D. J. Discovery of new biocatalysts for the glycosylation of terpenoid scaffolds. *Chemistry* **14**, 6656–6662 (2008).
7. Lim, E.-K., Ashford, D. A., Hou, B., Jackson, R. G. & Bowles, D. J. Arabidopsis glycosyltransferases as biocatalysts in fermentation for regioselective synthesis of diverse quercetin glucosides. *Biotechnol. Bioeng.* **87**, 623–631 (2004).
8. Lim, E.-K. *et al.* The activity of Arabidopsis glycosyltransferases toward salicylic acid, 4-hydroxybenzoic acid, and other benzoates. *J. Biol. Chem.* **277**, 586–592 (2002).
9. Poppenberger, B. *et al.* The UGT73C5 of Arabidopsis thaliana glucosylates brassinosteroids. *Proc. Natl. Acad. Sci. U. S. A.* **102**, 15253–15258 (2005).
10. Noguchi, A. *et al.* A UDP-glucose:Isoflavone 7-O-glucosyltransferase from the roots of soybean (Glycine max) seedlings. *J. Biol. Chem.* **282**, 23581–23590 (2007).
11. Jugdé, H., Nguy, D., Moller, I., Cooney, J. M. & Atkinson, R. G. Isolation and characterization of a novel glycosyltransferase that converts phloretin to phlorizin, a potent antioxidant in apple. *FEBS J.* **275**, 3804–3814 (2008).
12. Hou, B., Lim, E.-K., Higgins, G. S. & Bowles, D. J. N-glucosylation of cytokinins by glycosyltransferases of Arabidopsis thaliana. *J. Biol. Chem.* **279**, 47822–47832 (2004).
13. Hansen, K. S. *et al.* The in vitro substrate regiospecificity of recombinant UGT85B1, the cyanohydrin glucosyltransferase from Sorghum bicolor. *Phytochemistry* **64**, 143–151 (2003).
14. Jing, T. *et al.* UGT85A53 promotes flowering via mediating abscisic acid glucosylation and FLC transcription in Camellia sinensis. *J. Exp. Bot.* **71**, 7018–7029 (2020).
15. Olsson, K. *et al.* Microbial production of next-generation stevia sweeteners. *Microb. Cell Fact.* **15**, 207 (2016).
16. Lim, E.-K., Higgins, G. S., Li, Y. & Bowles, D. J. Regioselectivity of glucosylation of caffeic acid by a UDP-glucose:glucosyltransferase is maintained in planta. *Biochem. J.* **373**, 987–992 (2003).
17. Jackson, R. G. *et al.* Identification and biochemical characterization of an Arabidopsis indole-3-acetic acid glucosyltransferase. *J. Biol. Chem.* **276**, 4350–4356 (2001).
18. Záveská Drábková, L., Honys, D. & Motyka, V. Evolutionary diversification of cytokinin-specific glucosyltransferases in angiosperms and enigma of missing cis-zeatin O-glucosyltransferase gene in Brassicaceae. *Sci. Rep.* **11**, 7885 (2021).
19. Rai, A., Smita, S. S., Singh, A. K., Shanker, K. & Nagegowda, D. A. Heteromeric and homomeric geranyl diphosphate synthases from Catharanthus roseus and their role in monoterpene indole alkaloid biosynthesis. *Mol. Plant* **6**, 1531–1549 (2013).
20. Simkin, A. J. *et al.* Characterization of the plastidial geraniol synthase from Madagascar periwinkle which initiates the monoterpenoid branch of the alkaloid pathway in internal phloem associated parenchyma. *Phytochemistry* **85**, 36–43 (2013).
21. Collu, G. *et al.* Geraniol 10-hydroxylase, a cytochrome P450 enzyme involved in terpenoid indole alkaloid biosynthesis. *FEBS Lett.* **508**, 215–220 (2001).
22. Miettinen, K. *et al.* The seco-iridoid pathway from Catharanthus roseus. *Nature Communications* vol. 5 (2014).
23. Krithika, R. *et al.* Characterization of 10-hydroxygeraniol dehydrogenase from Catharanthus roseus reveals cascaded enzymatic activity in iridoid biosynthesis. *Sci. Rep.* **5**, 8258 (2015).

24. Geu-Flores, F. *et al.* An alternative route to cyclic terpenes by reductive cyclization in iridoid biosynthesis. *Nature* **492**, 138–142 (2012).
25. Chalfie, M., Tu, Y., Euskirchen, G., Ward, W. W. & Prasher, D. C. Green Fluorescent Protein as a Marker for Gene Expression. *Science* **263**, 802–805 (1994).
26. Reed, J. *et al.* A translational synthetic biology platform for rapid access to gram-scale quantities of novel drug-like molecules. *Metab. Eng.* **42**, 185–193 (2017).
27. Schmidt, A. *et al.* A bifunctional geranyl and geranylgeranyl diphosphate synthase is involved in terpene oleoresin formation in Picea abies. *Plant Physiol.* **152**, 639–655 (2010).
28. Lichman, B. R. *et al.* The evolutionary origins of the cat attractant nepetalactone in catnip. *Sci. Adv.* **6**, eaba0721 (2020).
29. Lichman, B. R. *et al.* Uncoupled activation and cyclization in catmint reductive terpenoid biosynthesis. *Nat. Chem. Biol.* **15**, 71–79 (2019).
30. Salim, V., Wiens, B., Masada-Atsumi, S., Yu, F. & De Luca, V. 7-deoxyloganetic acid synthase catalyzes a key 3 step oxidation to form 7-deoxyloganetic acid in Catharanthus roseus iridoid biosynthesis. *Phytochemistry* **101**, 23–31 (2014).
31. Asada, K. *et al.* A 7-deoxyloganetic acid glucosyltransferase contributes a key step in secologanin biosynthesis in Madagascar periwinkle. *Plant Cell* **25**, 4123–4134 (2013).
32. Murata, J., Roepke, J., Gordon, H. & De Luca, V. The leaf epidermome of Catharanthus roseus reveals its biochemical specialization. *Plant Cell* **20**, 524–542 (2008).
33. Irmler, S. *et al.* Indole alkaloid biosynthesis in Catharanthus roseus: new enzyme activities and identification of cytochrome P450 CYP72A1 as secologanin synthase. *Plant J.* **24**, 797–804 (2008).
34. De Luca, V., Marineau, C. & Brisson, N. Molecular cloning and analysis of cDNA encoding a plant tryptophan decarboxylase: comparison with animal dopa decarboxylases. *Proc. Natl. Acad. Sci. U. S. A.* **86**, 2582–2586 (1989).
35. Pasquali, G. *et al.* Coordinated regulation of two indole alkaloid biosynthetic genes from Catharanthus roseus by auxin and elicitors. *Plant Mol. Biol.* **18**, 1121–1131 (1992).
36. Yauk, Y.-K. *et al.* Manipulation of flavour and aroma compound sequestration and release using a glycosyltransferase with specificity for terpene alcohols. *Plant J.* **80**, 317–330 (2014).
37. Ohgami, S. *et al.* Volatile glycosylation in tea plants: Sequential glycosylations for the biosynthesis of aroma β-primeverosides are catalyzed by two Camellia sinensis glycosyltransferases. *Plant Physiol.* **168**, 464–477 (2015).
38. Nagatoshi, M., Terasaka, K., Nagatsu, A. & Mizukami, H. Iridoid-specific glucosyltransferase from Gardenia jasminoides. *J. Biol. Chem.* **286**, 32866–32874 (2011).
39. Jones, P. R., Møller, B. L. & Høj, P. B. The UDP-glucose:P-hydroxymandelonitrile-O-glucosyltransferase that catalyzes the last step in synthesis of the cyanogenic glucoside dhurrin in sorghum bicolor. *J. Biol. Chem.* **274**, 35483–35491 (1999).
40. Bönisch, F. *et al.* A UDP-glucose:Monoterpenol glucosyltransferase adds to the chemical diversity of the grapevine metabolome. *Plant Physiol.* **165**, 561–581 (2014).
41. Chen, B. *et al.* Dynamic imaging of genomic loci in living human cells by an optimized CRISPR/Cas system. *Cell* **155**, 1479–1491 (2013).
