# Supplementary material for: Reconstitution of monoterpene indole alkaloid biosynthesis in genome engineered Nicotiana benthamiana
Source: Commun Biol. 2022 Sep 10;5:949. doi: 10.1038/s42003-022-03904-w (PMC9464250; doi:10.1038/s42003-022-03904-w)
Supplement: Supplementary file 2 — Description of Additional Supplementary Data [file 42003_2022_3904_MOESM2_ESM.pdf]

Description of Additional Supplementary Files

File name: Supplementary Data 1

Description: Numerical source data for graphs in Figures 3, 5, 6 and Supplementary Figures 6, 7 and 13
