# Supplementary material for: Reconstitution of monoterpene indole alkaloid biosynthesis in genome engineered Nicotiana benthamiana
Source: Commun Biol. 2022 Sep 10;5:949. doi: 10.1038/s42003-022-03904-w (PMC9464250; doi:10.1038/s42003-022-03904-w)
Supplement: Supplementary file 4 — Reporting Summary [file 42003_2022_3904_MOESM4_ESM.pdf]

nature portfolio

Corresponding author(s): PATRON,NJ

Last updated by author(s): Jul 29, 2022

# Reporting Summary

Nature Portfolio wishes to improve the reproducibility of the work that we publish. This form provides structure for consistency and transparency in reporting. For further information on Nature Portfolio policies, see our Editorial Policies and the Editorial Policy Checklist.

## Statistics

For all statistical analyses, confirm that the following items are present in the figure legend, table legend, main text, or Methods section.

| n/a | Confirmed | |
|---|---|---|
| ☐ | ☒ | The exact sample size (*n*) for each experimental group/condition, given as a discrete number and unit of measurement |
| ☐ | ☒ | A statement on whether measurements were taken from distinct samples or whether the same sample was measured repeatedly |
| ☒ | ☐ | The statistical test(s) used AND whether they are one- or two-sided<br>*Only common tests should be described solely by name; describe more complex techniques in the Methods section.* |
| ☒ | ☐ | A description of all covariates tested |
| ☒ | ☐ | A description of any assumptions or corrections, such as tests of normality and adjustment for multiple comparisons |
| ☒ | ☐ | A full description of the statistical parameters including central tendency (e.g. means) or other basic estimates (e.g. regression coefficient) AND variation (e.g. standard deviation) or associated estimates of uncertainty (e.g. confidence intervals) |
| ☒ | ☐ | For null hypothesis testing, the test statistic (e.g. *F*, *t*, *r*) with confidence intervals, effect sizes, degrees of freedom and *P* value noted<br>*Give P values as exact values whenever suitable.* |
| ☒ | ☐ | For Bayesian analysis, information on the choice of priors and Markov chain Monte Carlo settings |
| ☒ | ☐ | For hierarchical and complex designs, identification of the appropriate level for tests and full reporting of outcomes |
| ☒ | ☐ | Estimates of effect sizes (e.g. Cohen's *d*, Pearson's *r*), indicating how they were calculated |

*Our web collection on statistics for biologists contains articles on many of the points above.*

## Software and code

Policy information about availability of computer code

| | |
|---|---|
| Data collection | No software was used to collect data. |
| Data analysis | All analysis was done using available (non-custom) code.<br>Transcriptome analysis: Data analysis was performed using a web-based instance of Galaxy. Short reads from RNA-seq were mapped to the publicly available N. benthamiana genome using hisat2 v2.1.0 default parameters. Assembled transcripts were generated using Stringtie v1.3.3.1. Transcripts were consolidated using Stringtie merge v1.3.3. Short reads were aligned to the expanded genome using hisat2 v2.1.0. Differential expression tables were generated using DESeq2 v2.11.40.1. Transcripts from the merged transcriptome were translated using transdecoder and the longest coding sequences annotated using phmmer v3.1v2 with the Swiss-Prot protein database (accessed May 2018) as reference.<br>Phylogenetic analysis: GenBank accession numbers of the sequences analyzed are provided in Supplementary Table 5. The 193 sequences were aligned using MUSCLE 3.8.425 (Edgar 2004) and a phylogenetic tree with 100 bootstraps was generated using RAxML version 8.2.11 |

For manuscripts utilizing custom algorithms or software that are central to the research but not yet described in published literature, software must be made available to editors and reviewers. We strongly encourage code deposition in a community repository (e.g. GitHub). See the Nature Portfolio guidelines for submitting code & software for further information.

# Data

Policy information about availability of data

All manuscripts must include a data availability statement. This statement should provide the following information, where applicable:

- Accession codes, unique identifiers, or web links for publicly available datasets
- A description of any restrictions on data availability
- For clinical datasets or third party data, please ensure that the statement adheres to our policy

Sequences (and DNA samples) of plasmids have been deposited in Addgene. Deposit numbers are 177019 - 177092 and are provided for each plasmid in Supplementary Tables. Transcriptome data has been deposited in the NCBI Sequence Read Archive database under project ID PRJNA841421.

# Human research participants

Policy information about studies involving human research participants and Sex and Gender in Research.

| | |
|---|---|
| Reporting on sex and gender | This study does not involved human research participants |
| Population characteristics | *Describe the covariate-relevant population characteristics of the human research participants (e.g. age, genotypic information, past and current diagnosis and treatment categories). If you filled out the behavioural & social sciences study design questions and have nothing to add here, write "See above."* |
| Recruitment | *Describe how participants were recruited. Outline any potential self-selection bias or other biases that may be present and how these are likely to impact results.* |
| Ethics oversight | *Identify the organization(s) that approved the study protocol.* |

Note that full information on the approval of the study protocol must also be provided in the manuscript.

# Field-specific reporting

Please select the one below that is the best fit for your research. If you are not sure, read the appropriate sections before making your selection.

☒ Life sciences ☐ Behavioural & social sciences ☐ Ecological, evolutionary & environmental sciences

For a reference copy of the document with all sections, see nature.com/documents/nr-reporting-summary-flat.pdf

# Life sciences study design

All studies must disclose on these points even when the disclosure is negative.

| | |
|---|---|
| Sample size | A minimum of n=3 biological replicates were used for transcriptome analysis, assessment of derivative peaks in mutated plants and quantification of yield. Key experiments (i.e., quantification of 7-DLA and strictosidine) were repeated such that n=6 or n=10. |
| Data exclusions | No data was excluded |
| Replication | Key experiments (i.e., quantification of 7-DLA and strictosidine), each with n=3 biological replicates were repeated such that n=6 or n=10. |
| Randomization | The arrangement of samples agroinfiltrated into leaves was randomized across leaves and across plants in a given experiment. |
| Blinding | For identification of derivatives and quantification of metabolite products the experimental design, leaf infiltration and sample collection was performed by QMD (at the Earlham Institute). Samples were then blindly analyzed by LC (MPI Jena) using by UPLC/MS qTOF. |

# Reporting for specific materials, systems and methods

We require information from authors about some types of materials, experimental systems and methods used in many studies. Here, indicate whether each material, system or method listed is relevant to your study. If you are not sure if a list item applies to your research, read the appropriate section before selecting a response.

## Materials & experimental systems

| n/a | Involved in the study |
|---|---|
| ☒ | ☐ Antibodies |
| ☒ | ☐ Eukaryotic cell lines |
| ☒ | ☐ Palaeontology and archaeology |
| ☒ | ☐ Animals and other organisms |
| ☒ | ☐ Clinical data |
| ☒ | ☐ Dual use research of concern |

## Methods

| n/a | Involved in the study |
|---|---|
| ☒ | ☐ ChIP-seq |
| ☒ | ☐ Flow cytometry |
| ☒ | ☐ MRI-based neuroimaging |
